# Different Proteostasis Mechanisms Facilitate the Assembly of Individual Components on the Chitin Synthase 3 Complex at the Endoplasmic Reticulum

**DOI:** 10.3390/jof11030221

**Published:** 2025-03-14

**Authors:** Noelia Sánchez, Rosario Valle, César Roncero

**Affiliations:** Instituto de Biología Funcional y Genómica (IBFG) and Departamento de Microbiología y Genética, CSIC-Universidad de Salamanca, 37007 Salamanca, Spain; nsn@usal.es (N.S.); rrv@usal.es (R.V.)

**Keywords:** chitin synthase, endoplasmic reticulum, protein traffic

## Abstract

Chitin synthase 3 complex assembly begins at the endoplasmic reticulum where the formation of a Chs3/Chs7 complex facilitates its exit from the ER and its transport along the secretory route. In the present study, our work shows that orphan molecules of Chs7 can exit the ER and are later recycled from the early Golgi by coat protein I (COPI) machinery via the adaptor complex Erv41/Erv46. Moreover, an eventual excess of the protein in the Golgi is recognized by the GGA complex and targeted to the vacuole for degradation through the ESCRT machinery. Non-oligomerizable versions of Chs3 can also exit the ER individually and follow a similar route to that of Chs7. We therefore demonstrate the traffic of unassembled CS3 subunits and describe the cellular mechanisms that guarantee the correct assembly of this protein complex at the ER while providing a default traffic route to the vacuole in case of its failure. This traffic route is shared with canonical ER adaptors, such as Erv29 and Erv14, and other components of protein complexes. The comparative analysis of their traffic allows us to discern a cellular program that combines COPI recycling, proteasomal degradation, and vacuolar disposal for maintaining protein homeostasis at the ER.

## 1. Introduction

Proteins destined to the secretory pathway are translocated across the endoplasmic reticulum (ER) membrane where they fold and can undergo further post-translational modifications, proteolytic processing, oligomerization, and/or assembly into multi-subunit complexes. Since all of these processes are inherently error-prone, the cell has developed multiple quality control (QC) systems in different compartments to monitor and selectively retain, aggregate, or degrade those proteins that fail to fold, oligomerize, and/or assemble correctly (reviewed in [1]). These systems are essential for maintaining protein homeostasis in the cell (proteostasis), which is crucial for ensuring proper cellular function and preventing the onset of various diseases.

Although the role of protein misfolding has been extensively studied, further research is needed regarding the processes of oligomerization and protein complex assembly, especially considering the large number of protein complexes and their vital biological functions in eukaryotic cells. Indeed, previous analyses of genetic disorders have revealed that many disease-causing mutations affect complex assembly rather than protein folding [2,3] and that the formation of protein complexes becomes progressively impaired through stress and aging [4]. However, this field of research remains significantly challenging. For protein complexes to form, newly synthesized subunits must come together spatially and temporally and their assembly must occur stoichiometrically without generating potentially cytotoxic intermediates [5]. Therefore, if the assembly of protein complexes fails or there is a nonstoichiometric synthesis of the different subunits, the cell generate a continuous amount of orphan proteins and defective protein complexes. In fact, it has been estimated that 10% of the nascent mammalian proteome arises from nonstoichiometric synthesis or failed assembly (reviewed in [6]), which eventually can contribute to proteotoxicity or damage to various cellular structures (reviewed in [5,7]).

The role of the ubiquitin–proteasome system (UPS) in the quality control of monomeric proteins is well established (reviewed by [6,7]), and defects in this pathway have been linked to cytotoxicity [8]. Recently, the UPS, through ER-associated degradation (ERAD) or inner-nuclear membrane-associated degradation (INMAD), has been shown to distinguish between and selectively target the monomeric and unassembled forms of certain substrates for degradation by the proteasome (reviewed in [6,9]). Additionally, various assembly factors or proteases can ensure that complexes only incorporate appropriate subunits [10]. Moreover, a role for autophagy in eliminating defective complexes has also been proposed (reviewed in [11]). In the case of the orphan subunits of the fatty acid synthase (FAS) complex, the subunits Fas1 or Fas2 can be degraded by different QC systems, with the orphan Fas2 being targeted to a Ubr1-linked ubiquitin–proteasome pathway for elimination. By contrast, the orphan Fas1 is directed to the vacuole for degradation via bulk autophagy [12]. However, many orphan subunits or defective complexes manage to evade ERAD or INMAD and reach the early Golgi where they are recognized by the Golgi quality control (GQC) system. Once recognized in the early Golgi, they are recycled back to the ER for refolding or degradation through ERAD or INMAD. In yeasts, Rer1 is a major adaptor at the Golgi that recognizes ER resident proteins as well as misfolded proteins or unassembled subunits through their transmembrane domains for retrieval from the early Golgi to the ER via the COPI-mediated retrograde transport pathway (reviewed in [13]). If this recycling process becomes saturated, orphan proteins or defective complexes may proceed through the Golgi and be directed to the vacuole for ultimate degradation (reviewed by [1]).

Chs3 is a polytopic protein involved in chitin synthesis in yeasts (reviewed in [14]). Its exit from the ER depends on the action of the specific chaperone Chs7 [15], which plays a similar role to Shr3, Gsf2 and Pho86 for preventing the transmembrane mismatching of their substrates, amino acid permeases (AAPs), Hxt1-2, and Pho84, respectively, during their insertion into and folding at the ER membrane [16]. In addition, Chs3 oligomerizes in this compartment, and defects in this process mean that non-oligomerized proteins can be recognized in the early Golgi by the Rer1 receptor and recycled to the ER via COPI vesicles [17]. A potential excess of unfolded Chs3 at the ER can be alleviated by the degradation of the protein by the ERAD and INMAD systems [18]. However, it has recently been shown that Chs7 interacts with Chs3 to form a complex that traffics to the plasma membrane (PM) [19]. This interaction remains possible even with the non-oligomerizable forms of Chs3 and allows the formation of functional complexes capable of reaching the PM, fulfilling their biological role in chitin synthesis [17].

The revelation that Chs3 forms a complex with Chs7 and its ability to oligomerize in the ER positions Chs3 as an excellent model for studying quality control systems that regulate the proper formation of protein complexes along the secretory pathway. This study addresses the quality control mechanisms that monitor Chs3/Chs7 complex formation by studying the trafficking of their orphan subunits in this pathway. Our findings suggest that, beyond COPI recycling, unassembled subunits of CS3 can be eliminated by both proteasomal and vacuolar degradation depending on the intrinsic properties of the proteins. Additionally, our work on Chs7 has uncovered the potential cellular mechanisms responsible for the disposal of multiple ER multispanning membrane sorting receptors. These findings underscore the crucial role of vacuolar degradation in eliminating orphan subunits and defective complexes, thereby contributing directly to protein homeostasis in yeast cells.

## 2. Materials and Methods

**Yeast strains and growth conditions.** All *S. cerevisiae* strains used in this work are listed in Table S1. All the experiments were carried out in the same genetic background for every protein, W303 or BY4741/BY4742, depending on the case.

Competent yeast cells were transformed using the lithium acetate/polyethylene glycol procedure [20] with the pertinent plasmids or polymerase chain reaction (PCR) fragments (cassettes). Gene deletions were made by a PCR-mediated gene replacement technique, using different deletion cassettes based on the resistance genes *natMX4*, *kanMX4,* and *hphNT1* [21], or by the ability to synthesize a specific amino acid like *HIS3* or *URA3*.

Tagging in the genome with GFP, superfolderGFP (sfGFP), mCherry, or the VENUS fragments (VC and VN) of the different proteins was carried out in their C-terminal region by homologous recombination. The tags were previously obtained from the cast plasmids (see Table S2) after PCR amplification together with the marker, which later allowed the selection of the yeast transformants containing the corresponding insert. The correct insertion of the tag was always verified by PCR.

Yeast cells were cultured in rich YEPD medium (1% *w/v* yeast extract, 2% *w/v* peptone, and 2% *w/v* dextrose) or in synthetic dextrose medium (2% *w/v* dextrose and 0.67% *w/v* yeast nitrogen base) supplemented with 0.2% (*w*/*v*) of an amino acid mixture lacking specific amino acid(s) when plasmid selection was necessary. All yeast strains were grown at 28 °C, except the thermosensitive mutants *sec23* and *sec31-1,* which were grown at 25 °C as the permissive temperature and 37 °C as the restrictive temperature.

**Construction of plasmids.** The different plasmids used in this work are described in Table S2. The plasmid pRS315::^Δ126^*CHS3*^1-490^-GFP was obtained by homologous recombination in vivo in three steps. First, the pRS315::^Δ126^*CHS3*-GFP plasmid was linearized with the CspCI enzyme followed by a klenow treatment to generate blunt ends. Second, a DNA fragment was amplified by PCR using specific primers and the pRS315::*CHS3*^1-490^-GFP plasmid as a template. Third, the yeast strain was transformed with both constructs and the corresponding transformants were selected in SD media. The DNA from different independent transformants was recovered by electroporation in *E. coli*, purified, and sequenced to confirm the bona fide construction.

**Fluorescence microscopy.** Yeast cells expressing GFP-tagged proteins were grown to a logarithmic phase in SD medium supplemented with 0.2% adenine. Living cells were visualized directly by fluorescence microscopy. Images were obtained using a Nikon 90i epifluorescence microscope (100× objective, Melville, NY, USA; NA: 1.45) equipped with a Hamamatsu ORCA ER digital camera and a specific Chroma filter in each case: GFP (49002 ET-GFP), YFP (49003 ET-YFP) or mCherry (49005 ET-DsRed) (TRITC/Cy3) (Chroma Technology Corp, Bellows Falls, VT, USA). Images were obtained and processed using Metamorph Premier 7.1.2 software. For the endocytosis inhibition experiments, the cells were treated with the actin-depolymerizing drug, latrunculin A, dissolved in DMSO at a final concentration of 30 µM in the respective medium [22]. The cells were later incubated at 28 °C and the samples were visualized during the 60 min treatment.

**BiFC (bi-molecular fluorescence complementation).** In these studies, the interaction between two proteins tagged with the N-terminal (VN) and C-terminal (VC) fragments of the fluorescent protein VENUS was used as an indicator of the potential interaction between the proteins [23]. Visualization was performed in the YFP channel (49003 ET-YFP) since the VENUS protein is a variant of the YFP fluorophore (λA/λE, 515/528 nm).

**Protein extracts and immunoblotting.** A protocol based on the use of trichloroacetic acid (TCA) [24] was carried out to process the samples for Western blot analysis. Proteins were extracted using an equal number of cells from logarithmically growing cultures. Cells were centrifuged, washed in 20% TCA, and then disrupted in 1.5 mL tubes with 50 μL of 20% TCA and glass beads (0.45 mm, Sigma, Kawasaki, Japan) during three pulses of 30 s each with an intensity of 5.5 in a Fast Prep shaker (FP120 Bio101, Savant, Cleveland, OH, USA). Lysates were diluted with 400 μL of 5% TCA, and after vortexing, the beads were removed by centrifugation. The supernatants were centrifuged for 10 min at 800 *g*, and the pellets were resuspended in a 100 μL mix (containing 20 μL of 10% sodium dodecyl sulfate [SDS], 20 μL of water, and 60 μL of 1 M Tris base) and incubated at 95 °C for 5 min. Samples were centrifuged for 2 min at 15,700 *g*, and the supernatants were collected. The protein concentration was measured using the Bradford method [25]. Fifty microliters of each protein sample were resuspended in 50 μL of 2× sample buffer (100 mmol/L Tris-HCl pH 6.8, 4% SDS, 20% glycerol, 25 mmol/L dithiothreitol [DTT], and traces of bromophenol blue) by vortexing, and between 50 and 100 μg of protein was loaded per lane for Western blot analysis. All steps of the protocol were performed at 4 °C to avoid protein degradation.

Extracts were separated in SDS-PAGE gels with different acrylamide concentrations (6.5–10%) depending on the size of the protein under study. Separated proteins were transferred to polyvinylidene difluoride (PVDF) membranes, which were later blocked with 1× Tris-buffered saline with 0.1% Tween 20 (TBS-T, Sigma) supplemented with 3% nonfat milk for 1 h. Then, the membranes were incubated with the corresponding antibodies in 1× TBST with 3% nonfat milk overnight at 4 °C: mouse monoclonal anti-GFP JL-8 (Living Colors, Clontech, Mountain View, CA, USA; 1:1000 dilution), mouse monoclonal anti-HA (12CA5, Roche; 1:5000 dilution), mouse monoclonal anti-Myc (9E10, Santa Cruz Biotechnology, Dallas, TX, USA; 1:5000 dilution), mouse monoclonal anti-Tubulin (T5162, Sigma; 1:5000 dilution) or mouse monoclonal anti-actin (JLA20, Sigma; 1:2500 dilution). After washing three times (10 min each), the membranes were incubated for 1 h with the secondary antibody (polyclonal anti-mouse or anti-rabbit conjugated with horseradish peroxidase, 1:5000 dilution in 1× TBST with 3% nonfat milk). After three washes with 1× TBST (10 min each), the blots were developed using the ECL Kit (Advansta, San Jose, CA, USA). Proteins detected by the antibodies were visualized in a *FUSION FX* device (Vilber, Collégien, France).

**Co-immunoprecipitation.** For these experiments, cells from 100 mL of logarithmic cultures were harvested by centrifugation and washed with cold water. Cells were mechanically broken in 200 μL of lysis buffer (50 mM Tris-HCl pH 8.0, 0.1% Triton, 150 mM NaCl), containing 1× protease inhibitor cocktail (1 mM PMSF, 1 μg/μL aprotinin, and 1 μg/μL leupeptin), using glass beads during three pulses of 32 s each with an intensity of 5.5 in a Fast Prep shaker (FP120 Bio101, Savant). The lysates were later cleared of cell debris (16,000× *g* for 5 min at 4 °C). Then, 2.5 mg of total protein from these lysates was diluted to 400 μL with IP buffer (50 mM Tris-HCl pH 8.0, 0.1% Triton, 150 mM NaCl, and 2 mg/mL bovine serum albumin). This suspension was incubated in an orbital rotor at 4 °C for 3.5 h with the corresponding antibody: mouse monoclonal anti-Myc antibody (9E10, Santa Cruz Biotechnology) diluted 1:75 to immunoprecipitate Chs7-13xMyc or rabbit polyclonal anti-GFP antibody (Invitrogen, Waltham, MA, USA) diluted 1:100 in the case of Erv14-mCi. Later the suspension was mixed with 50 μL of 0.1 g/mL of Protein A Sepharose (GE Healthcare, Chicago, IL, USA) and incubated overnight at 4 °C. The beads were washed three times with lysis buffer and boiled with 4x SDS loading buffer for 5 min. To visualize the Myc or HA tags, 10–12 μL of each sample was separated in SDS-PAGE gels with 7.5% acrylamide to see the Chs3-3xHA constructs or with 11% acrylamide to see Chs7-13xMyc. To visualize the GFP or mCitrine tags, 25 μL of each sample was separated in SDS-PAGE gels with 11–13% acrylamide to see Gga2-GFP or Erv14-mCi. The separated proteins were subjected to immunoblotting as described above.

**Cross-linking assays.** To assess the potential aggregation of the constructs, cross-linking assays were performed using the DSP cross-linker (Pierce, Thermo, Waltham, MA, USA) according to Kota [16]. First, 30–50 mL of culture was harvested and resuspended in 200 μL of phosphate-buffered saline (PBS, pH 7.4). After breaking the cells with glass beads and removing the cell debris by centrifugation (15,700× *g* at 4 °C for 5 min), 10 μg of total protein was treated with DSP at the indicated concentrations for 30 min at 22 °C. Next, the samples were treated with 30 mM Tris-HCl (pH 7.5) for 30 min at 22 °C to neutralize free reactive groups. Control samples were also treated with 40 mM DTT for 30 min at 37 °C. Finally, all samples were boiled for 5 min at 95 °C with a loading buffer prepared without β-mercaptoethanol. Then, 10 μg of protein was separated on an SDS-PAGE gel containing 6.5% acrylamide.

**Quantifications and image designing.** Microscopy and Western blot image processing and quantification were performed using ImageJ-FIJI software (2.9.0/1.53t version, NIH). Western blot data were obtained in ImageJ (with the “Analyze Gels” tool) to be later analyzed in Microsoft Excel (16.68 version) and GraphPad Prism 9.4. Relative amounts (%) of the free-GFP/sfGFP band were calculated by dividing the intensity of the free-GFP/sfGFP band (~27 kDa) between the total intensity provided by the GFP signal (intensity of the free-GFP band plus the intensity of the protein-GFP/sfGFP band). Bar plots represent the average value of at least 3 experiments (*n* = 3) and SD is indicated by the error bars. Statistical significance was calculated always comparing against the data of the control strain in each case using an unpaired t-test with Welch’s correction. *p*-value notations: ns = no significative; * (*p* < 0.05); ** (*p* < 0.01); *** (*p* < 0.001); and **** (*p* < 0.0001). Relative amounts (%) of the GFP/sfGFP processing band (P), indicating proteasomal degradation, were obtained using the same approach used to obtain data for Figure S7B.

The bar plots were generated using GraphPad Prism 9.4. All the images presented in this work (microscopy, blots, and bar plots) were prepared using Adobe Photoshop CS5 software.

## 3. Results

**Chs7 and ^∆126^Chs3 traffic individually to the vacuole.** The major chitin synthase 3 follows a well-defined route from the ER to the PM where it exerts its function [14]. Later, the protein is endocyted and mostly recycled by the AP-1 adaptor to the trans-Golgi network (TGN) [26], while a small amount of it travels to the vacuole through the late endosomal compartment [27]. Chs7 was originally described as a dedicated ER chaperone required for the export of Chs3. However, recently, it has been shown that the Chs7 protein forms a stable complex with Chs3 [19] and follows it through the secretory route (Figure S1) to form the active chitin synthase III (CSIII) at the PM. This new vision of Chs7 challenges the previously reported role of Chs7 as an ER chaperone, which prompted us to directly investigate the traffic of Chs7.

**Figure 1:**
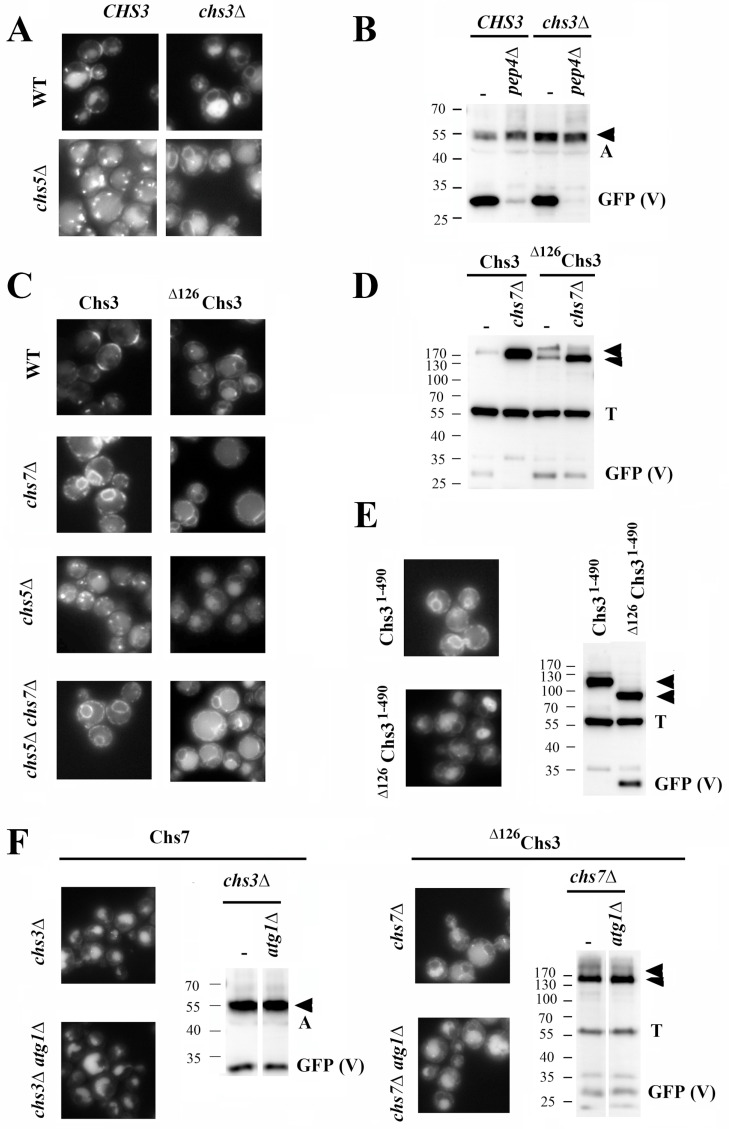
The individual traffic of CS3 components. (**A**) The localization of Chs7-sfGFP in wild-type (WT) and *chs5∆* strains depending on the presence of the Chs3 protein. (**B**) Western blot showing the vacuolar degradation of Chs7-sfGFP, depending on its interaction with Chs3, and the presence of the Pep4 protease as indicated. Note that the generation of the free-sfGFP band is dependent on Pep4 activity. (**C**) The localization of Chs3-GFP and ^∆126^Chs3-GFP proteins in the indicated strains. (**D**) Western blot showing the vacuolar degradation of Chs3-GFP and ^∆126^Chs3-GFP depending on the presence of Chs7. (**E**) The localization of Chs3^1-490^–GFP y ^∆126^Chs3^1-490^–GFP in the *chs3∆* mutant. Note the vacuolar localization and the appearance of the free-GFP band after the deletion of the N-terminal region. (**F**) The individual traffic of Chs7-sfGFP and ^∆126^Chs3-GFP to the vacuole is independent of Atg1. The localization of the indicated proteins and Western blots showing the free-GFP bands. Actin (A) or Tubulin (T) are used as loading controls in the Western blot analysis. GFP (V) refers to the free-GFP band and its intensity serves as a marker of vacuolar protein degradation.

In wild-type cells, Chs7 localized at the neck region, the trans-Golgi network/early endosome (TGN/EE) (chitosomes), and the vacuole (Figure 1A), colocalizing with Chs3 in all of these positions (Figure S1). This colocalization persisted after blocking the intracellular traffic of Chs3 at the Golgi in the *chs5∆* mutant (Figure 1A) and also in other trafficking mutants (Figure S1). When Chs7 localization was checked in the *chs3∆* mutant, the protein was found distributed between the ER and the vacuole, without apparently reaching the PM. This localization was not affected in the *chs5∆* mutant (Figure 1A). Moreover, the GFP band associated with the degradation of Chs7 in the vacuole (GFP (V)) was clearly visible, even in the absence of Chs3, and disappeared in the absence of the vacuolar protease Pep4 (Figure 1B). Altogether, these results indicate that Chs7 traffics to the vacuole independently of Chs3. Thus, a new potential traffic route for this protein was revealed.

A critical step in the traffic of Chs3 is its exit from the ER, which is strictly dependent on its interaction with the Chs7 chaperone [15] to facilitate its loading in COPII vesicles [16]. Chs7 also interacts with the non-oligomerized ^∆126^Chs3 protein, allowing its traffic to the PM and the synthesis of chitin [17]. Therefore, it was rather surprising to find a strong vacuolar signal for the ^∆126^Chs3 protein in the *chs7∆* mutant. By contrast, the wild-type Chs3 protein remained fully retained at the ER in this mutant (Figure 1C). The ^∆126^Chs3 protein was found in both the membrane and the lumen of the vacuoles, and its vacuolar degradation was confirmed by Western blot analysis and the appearance of a 27 KDa free-GFP band. The vacuolar degradation of the wild-type protein was not observed due to its complete retention at the ER in the *chs7∆* mutant (Figure 1D). Moreover, the deletion of the ∆126 N-terminal region from Chs3^1-490^, a truncated Chs3 protein that is fully retained in the ER in the presence of Chs7 (Figure 1E), also elicited its transport to the vacuole. This result confirmed the specific role of this N-terminal domain in preventing the traffic of Chs3 to the vacuole in the absence of Chs7. The vacuolar signal in the absence of Chs7 was also observed for ^∆15-140^Chs3 and ^∆26-125^Chs3 but not for the ^∆63^Chs3 or ^∆63-125^Chs3 proteins (Supplementary Figure S2A,B). These findings indicated that this traffic to the vacuole was directly associated with the absence of the previously described oligomerization domain of the Chs3 protein [17]. In the *chs7∆* mutant, the ^∆126^Chs3 protein was not functional, did not localize at the neck, and still showed strong vacuolar localization in the *chs5∆* mutant (Figure 1C). Taken together, this suggests that in the absence of Chs7, ^∆126^Chs3 traffics from the ER to the vacuole independently of its transit through the PM.

The individual traffic of these proteins to the vacuole could simply be mediated by the described autophagy of the ER (reviewed in [28]; therefore, we tested their traffic and vacuolar processing in the *atg1∆* mutant. As shown in Figure 1F, both Chs7 and ^∆126^Chs3 individual proteins were neatly localized in the vacuole and the vacuolar GFP band had the same intensity in the *atg1∆* mutant as in the control. These results discarded the arrival of Chs7/^∆126^Chs3 to the vacuole through autophagy, although a potential effect of microautophagy in this traffic cannot be discarded since the machinery involved in this process is still uncertain. These results suggest that Chs7 and ^∆126^Chs3 may exit the ER to reach the vacuole. Consequently, we then examined the role of COPII in this event.

**Figure 2:**
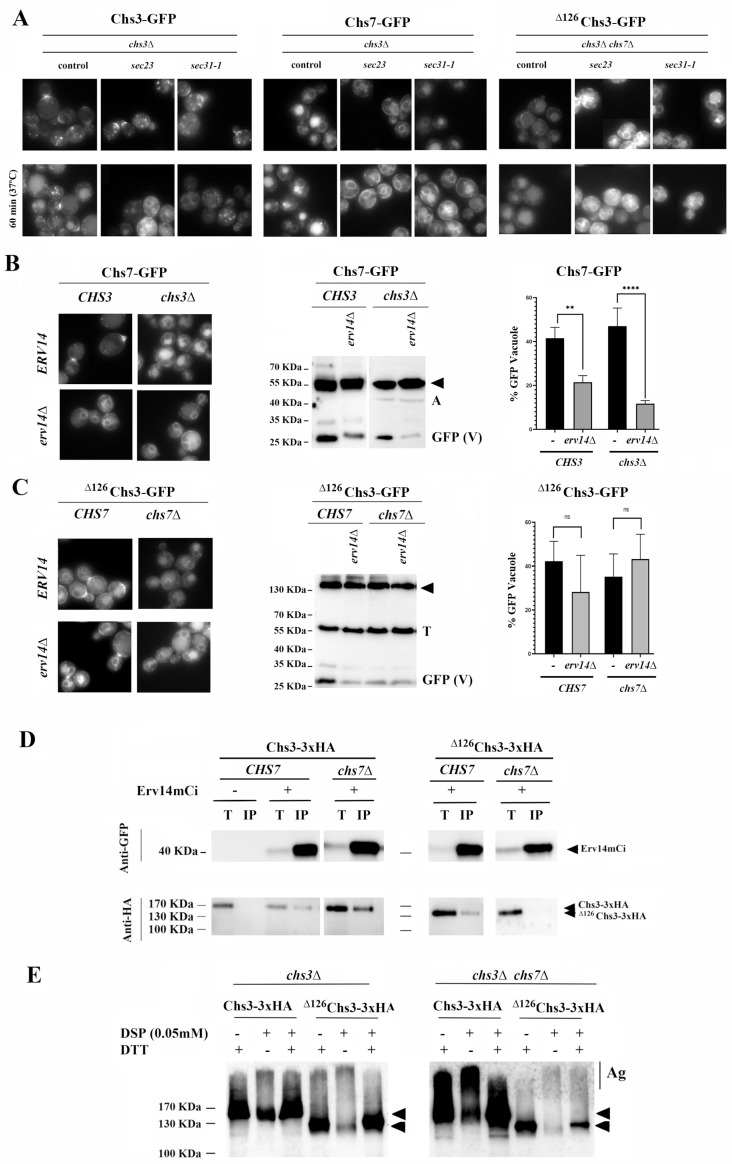
CS3 components individually exit the ER in COPII vesicles. (**A**) The localization of the indicated proteins in different *sec* mutants. Localization is assessed at 25 °C or after 60 min at 37 °C. The localization of WT Chs3-GFP is determined as the control (**left** panel). (**B**) The traffic of Chs7-sfGFP in *CHS3* and *chs3∆* strains is dependent on the Erv14 adaptor. Note the reduced vacuolar staining and the reduced levels of the free-GFP band in the *erv14∆* mutant. The **Right** panel presents the relative amounts (%) of the free-GFP band compared with the total amount of the Chs7-sfGFP in the indicated mutants. (**C**) The traffic of ^∆126^Chs3-GFP in *CHS7* and *chs7∆* strains is partially dependent on the Erv14 adaptor only in the presence of *CHS7*. Note the reduced vacuolar staining and the reduced levels of the free-GFP band in the *erv14∆* mutant in the *CHS7* strain but not in the *chs7∆* mutant. The **Right** panel presents the relative amounts (%) of the free-GFP band compared with the total amount of the ^∆126^Chs3-GFP in the indicated mutants. (**D**) Erv14-mCi coimmunoprecipitates with Chs3-3xHA independently of the presence of Chs7, but the coimmunoprecipitation of Erv14 with ^∆126^Chs3-3xHA is strongly reduced in the *chs7∆* mutant. Erv14-mCi is immunoprecipitated with antiGFP and the resulting immunoprecipitation products (IPs) are developed in Western blots with anti-GFP or anti-HA antibodies. Total extracts (T) are included as controls and the corresponding protein bands are marked with arrowheads. (**E**) DSP-mediated crosslinking experiments with Chs3-3xHA and ^∆126^Chs3-3xHA proteins in the presence or absence of Chs7 as indicated. Note the strong aggregation band (Ag) of the wild-type protein Chs3-3xHA in the *chs7∆* mutant, which does not appear in the case of the ^∆126^Chs3-3xHA protein. DTT treatment reverts the effect of DSP crosslinking and is used as a control. Arrowheads mark the monomeric forms of the proteins. Actin (A) or Tubulin (T) were used as the loading control in the Western blot analysis. GFP (V) refers to the free-GFP band and its intensity serves as a marker of vacuolar protein degradation. *p*-Values notation: ns = no significative; ** (*p* < 0.01) and **** (*p* < 0.0001).

**Chs7 and ^∆126^Chs3 exit the ER in COPII vesicles.** Chs7 is assumed to allow the exit of Chs3 from the ER in COPII vesicles as it has been described for the Gap1/Shr3 system [16]. Therefore, we tested the exit of Chs3, Chs7, and ^∆126^Chs3 from the ER in different COPII mutants (Figure 2). Figure 2A shows that growth at a restrictive temperature blocks the traffic of Chs3 to the PM in both *sec23* and *sec31-1* mutants as expected [29], confirming that the traffic of the Chs3/Chs7 complex is mediated by COPII. Moreover, a similar effect was observed for the Chs7 protein in the *chs3∆* mutant, in which the absence of Sec23 and Sec31 was associated with an increase in the ER signal and a reduction in vacuolar staining (Figure 2A, central panel). Similarly, the vacuolar staining of ^∆126^Chs3 in the *chs7∆* mutant was reduced at the restrictive temperature in both COPII mutants. Therefore, it can be concluded that orphan Chs7 and ^∆126^Chs3 proteins can exit the ER independently via COPII vesicles, which is the same for the Chs3 protein under wild-type conditions.

Chs3 trafficking relies on the ER adaptor Erv14 for its ER exit [17]. Accordingly, Chs7 was retained at the ER in the *erv14∆* mutant as part of the Chs7/Chs3 complex, and its traffic to the vacuole was reduced (Figure 2B). Similarly, the traffic of orphan Chs7 in the *chs3∆* mutant was reduced in the absence of Erv14. This led to its accumulation in the ER and its reduced traffic to the vacuole, as determined microscopically and by a less intense GFP band in Western blots (Figure 2B). In the *erv14∆* mutant, the amount of ^∆126^Chs3 reaching the vacuole in the presence of Chs7 was also reduced, concomitantly to its stronger retention at the ER (Figure 2C), confirming the role of Erv14 in the exit of the functional Chs3/Chs7 complexes from the ER [17]. However, the individual traffic of ^∆126^Chs3 in the *chs7∆* mutant was not affected by the absence of the adaptor since we did not observe a decrease in vacuolar staining or a reduction in the intensity of the GFP band (Figure 2C). Apparently, Erv14 directly facilitates the loading of Chs7 in COPII vesicles but not that of ^∆126^Chs3. Consequently, we examined how the presence of Chs7 affected the interaction of Chs3 with Erv14. Wild-type Chs3 coimmunoprecipitated with Erv14 independently of Chs7 (Figure 2D, left panel), suggesting a direct interaction between Chs3 and the adaptor. However, the coimmunoprecipitation of ^∆126^Chs3 with the adaptor was significantly reduced in the absence of Chs7. This result indicates that this protein poorly interacts with the Erv14 adaptor, which explains the limited role of this adaptor on the exit of individual molecules of ^∆126^Chs3 (Figure 2C).

Despite its direct interaction with Erv14, Chs3 remained retained in the ER in the *chs7∆* mutant because it aggregates in the absence of the ER chaperone (Figure 2E). The formation of aggregates has also been previously found for Gap1/Srh3 and Pho84/Pho86 [16] and is favored by the oligomerization of Chs3 through its N-terminal region [17]. Not surprisingly, ^∆126^Chs3 did not aggregate significantly in the *chs7∆* mutant (Figure 2E, compare the intensity of the aggregation bands) and was loaded into COPII vesicles. However, ^∆126^Chs3 interacted poorly with Erv14, and its loading is likely to have occurred through the ill-defined bulk-flow mechanism, which acts independently of dedicated adaptors [30].

**Figure 3:**
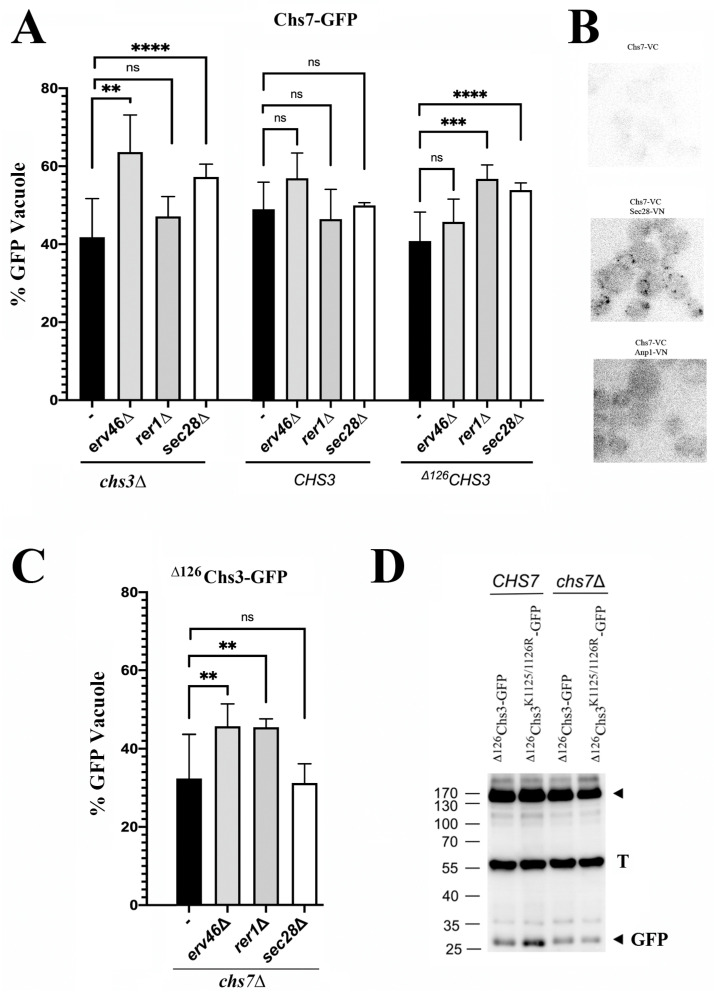
CS3 components are recycled to the ER in COPI vesicles. (**A**) Relative amounts (%) of the free-GFP band compared with the total amount of the Chs7-sfGFP in the indicated COPI or COPI-adaptor mutants. The assay is performed in a *chs3∆* background transformed with a pRS315 plasmid containing the indicated version of the Chs3 protein. (**B**) The BiFC of Chs7-VC with the Golgi markers Sec28-VN and Anp1-VN as indicated. (**C**) Relative amounts (%) of the free-GFP band compared with the total amount of the ^∆126^Chs3-GFP in the indicated COPI or COPI-adaptor mutants. The assay is performed in a *chs7∆* background. (**D**) A Western blot analysis of the indicated versions of the ^∆126^Chs3 protein in the presence or absence of Chs7. Bar plots represent the average value of at least 3 Western blot experiments (*n* = 3) and standard deviations (SDs) are indicated by the error bars. Statistical significance is calculated as indicated in Materials and Methods (MM). GFP (V) refers to the free-GFP band and its intensity serves as a marker of vacuolar protein degradation. *p*-Values notation: ns = no significative; ** (*p* < 0.01); *** (*p* < 0.001) and ****(*p* < 0.0001).

**Chs7 and ^∆126^Chs3 can be individually recycled from the Golgi by COPI.** The arrival of orphan molecules of Chs7 and ^∆126^Chs3 to the Golgi is an unexpected cellular event. Consequently, the cell would likely recognize them as incorrectly folded or unassembled and return them to the ER for proper folding or assembly. To test this hypothesis, we examined the potential return of these proteins to the ER by the COPI machinery. Figure 3A (left panel) shows that the arrival of individual Chs7 molecules to the vacuole is significantly increased in the *sec28∆* and *erv46∆* mutants as determined by the changes in the intensity of the GFP vacuolar band. This result highlights the retrograde transport of orphan Chs7 in COPI vesicles via the Erv41/46 adaptor complex [31]. Moreover, we observed a direct interaction of Chs7 with Sec28 (cis-Golgi) by bimolecular fluorescence complementation, but not with the Golgi marker Anp1 (Figure 3B). Interestingly, the traffic of Chs7 to the vacuole in the complex together with Chs3 was not affected in the COPI or COPI-adaptor mutants (Figure 3A, central panel), indicating that the Chs3/Chs7 complex traffics efficiently through the Golgi, precluding its recognition by the COPI machinery.

Previous studies showed that functional ^∆126^Chs3 is recycled by the COPI machinery through a di-lysine C-terminal domain in Chs3 [17]. Therefore, it was not surprising to find that the Chs7 trafficking to the vacuole, when forming a complex with ^∆126^Chs3, was also increased in several COPI and COPI-adaptor mutants (Figure 3A, right panel). When the recycling of the orphan ^∆126^Chs3 protein was examined, we observed that in the absence of Erv46 or Rer1, its traffic to the vacuole was significantly increased (Figure 3C), confirming its individual recycling by COPI through these adaptors. However, the traffic of orphan ^∆126^Chs3 to the vacuole was not increased after the deletion of the di-lysine motif (Figure 3D). Thus, its recycling by COPI seemed independent of the di-lysine motif, contrary to what was observed in the presence of Chs7 (Figure 3D and [17]).

Altogether, these results indicate that when arriving independently to the early Golgi, orphan Chs7 and ^∆126^Chs3 proteins are recognized as being unassembled and returned to the ER. Our results point to the participation of multiple mechanisms in the recruitment of these proteins by the COPI machinery at the early Golgi.

The results presented above not only highlight some of the rules governing the exit of Chs3 from the ER but also change our view on the role of Chs7 in this process. Previously, Chs7 has been described as an ER-dedicated chaperone. However, our results show that Chs7 also acts as a dedicated cargo adaptor that, when in excess, is returned from the Golgi to the ER to facilitate its interaction with its cargo. If this interaction does not occur, the protein would then be delivered to the vacuole for degradation as suggested by our initial observations (Figure 1). If our model is indeed correct, it would be tempting to speculate that other ER cargo adaptors may also follow a similar route (see below).

**Figure 4:**
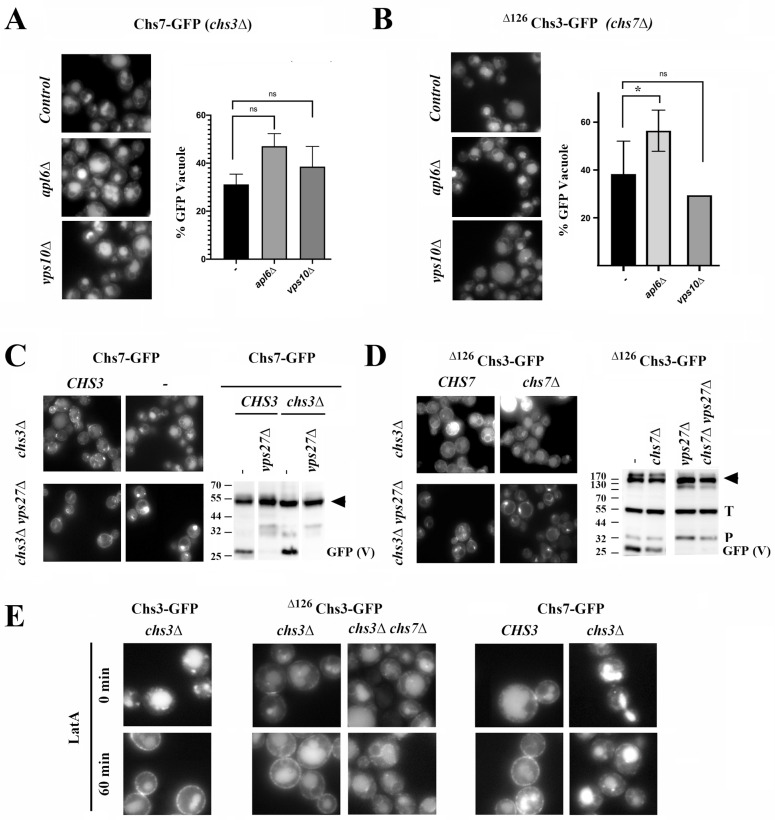
The sorting of CS3 components to the vacuole. (**A**) The localization of Chs7-sfGFP in the *chs3∆* background in the indicated mutants and bar plots showing the relative amounts of the free-sfGFP band in the same mutants. (**B**) The localization of ^∆126^Chs3-GFP in the *chs7∆* background in the indicated mutants and bar plots showing the relative amounts of the free-GFP band in the same mutants. (**C**) The microscopic localization and Western blot of Chs7-sfGFP in the ESCRT-0 *vps27∆* mutant in the presence or absence of Chs3 as indicated. Note how the accumulation of the protein at the E endosome is associated with a strong decrease in the free-sfGFP band. (**D**) The microscopic localization and Western blot of ^∆126^Chs3-GFP in the ESCRT-0 *vps27∆* mutants in the presence or absence of Chs7 as indicated. Note how the accumulation of the protein at the E endosome is associated with a strong decrease in the intensity of the free-GFP band. (**E**) The localization of the indicated proteins in the different strains before and after 60 min of incubation with the inhibitor of endocytosis latrunculin A (LatA). Bar plots represent the average values of at least 3 experiments (*n* = 3) and SD is indicated by the error bars in each case. Statistical significance was calculated as indicated in the MM. GFP (V) refers to the free-GFP band and its intensity serves as a marker of vacuolar protein degradation. *p*-Values notation: ns = no significative; * (*p* < 0.05).

**The route to the vacuole.** Next, we set out to examine how these proteins get to the vacuole. Since Chs7 travels together with Chs3 to the PM, it may later traffic to the vacuole through the endosomal compartment (Figure S1, [27]). However, conflicting results suggest that Chs3 may directly traffic from the TGN to the vacuoles via the AP-3 route [32], which Chs7 could also follow as part of the CS complex. To address the potential role of these routes in the individual traffic of Chs7, we analyzed its arrival to the vacuole microscopically and by measuring the amount of free-GFP in different mutants. Figure 4A shows how in the absence of AP-3 (*apl6∆*) or CPY (*vps10∆*) routes, the traffic of orphan Chs7 to the vacuole is not reduced. This result negates the potential involvement of these pathways in the individual traffic of Chs7 to the vacuole. Similarly, the individual traffic of ^∆126^Chs3 to the vacuole remained unhindered in the absence of AP-3 or CPY routes, based on the results obtained microscopically (Figure S4A) and the levels of free-GFP obtained in the Western blot analysis. These results discard the involvement of AP-3 and CPY routes in the traffic of these orphan proteins to the vacuole.

When we examined the potential role of endosomes in the individual traffic of Chs7 and ^∆126^Chs3 to the vacuole, we observed that vacuolar staining was significantly reduced in the *vps27∆* mutant. In addition, there was an accumulation of these proteins at the endosome E compartment (Figure 4C,D). Also, the processed GFP vacuolar band from both proteins was fully absent in this mutant (Figure 4C,D). The absence of the vacuolar t-SNARE Vam3 also reduced the traffic of these proteins to the vacuole (see Figure S4B). These results indicate that both Chs7 and ^∆126^Chs3 individually reach the vacuole through the late endosomal compartment, which is similar to what occurs in the case of functional Chs3/Chs7 complexes [27].

Although Chs3/Chs7 and ^∆126^Chs3/Chs7 complexes reached the vacuole after endocytosis from the PM [27], we have never observed a PM localization in the individual trafficking of ^∆126^Chs3 and Chs7 proteins. To confirm this finding, we inhibited the endocytic traffic from the PM by treating cells with latrunculin A (latA). In the presence of *CHS7*, latA treatment increased the amount of the Chs3 and ^∆126^Chs3 proteins visualized along the PM (Figure 4E) and, accordingly, with the formation of a functional Chs3/Chs7 complex, the localization of Chs7 along the PM was very similar (Figure 4E). Upon studying the individual traffic of ^∆126^Chs3 and Chs7, we did not observe the accumulation of either protein at the PM even after drug treatment; therefore, the transit of these orphan proteins through the PM is unlikely. In addition, the individual accumulation of both orphan proteins in the endosome E in the ESCRT mutant *vps27Δ* seems much stronger than their accumulation as part of the active complexes (Figure 4C,D; compare the intensity of the endosomal structures in the wild-type and mutant strains), suggesting the massive traffic of these proteins from the TGN directly to the vacuole. Altogether these results suggest that Chs7 and ^∆126^Chs3 proteins can be sorted individually at the TGN for their delivery to the vacuole.

**Figure 5:**
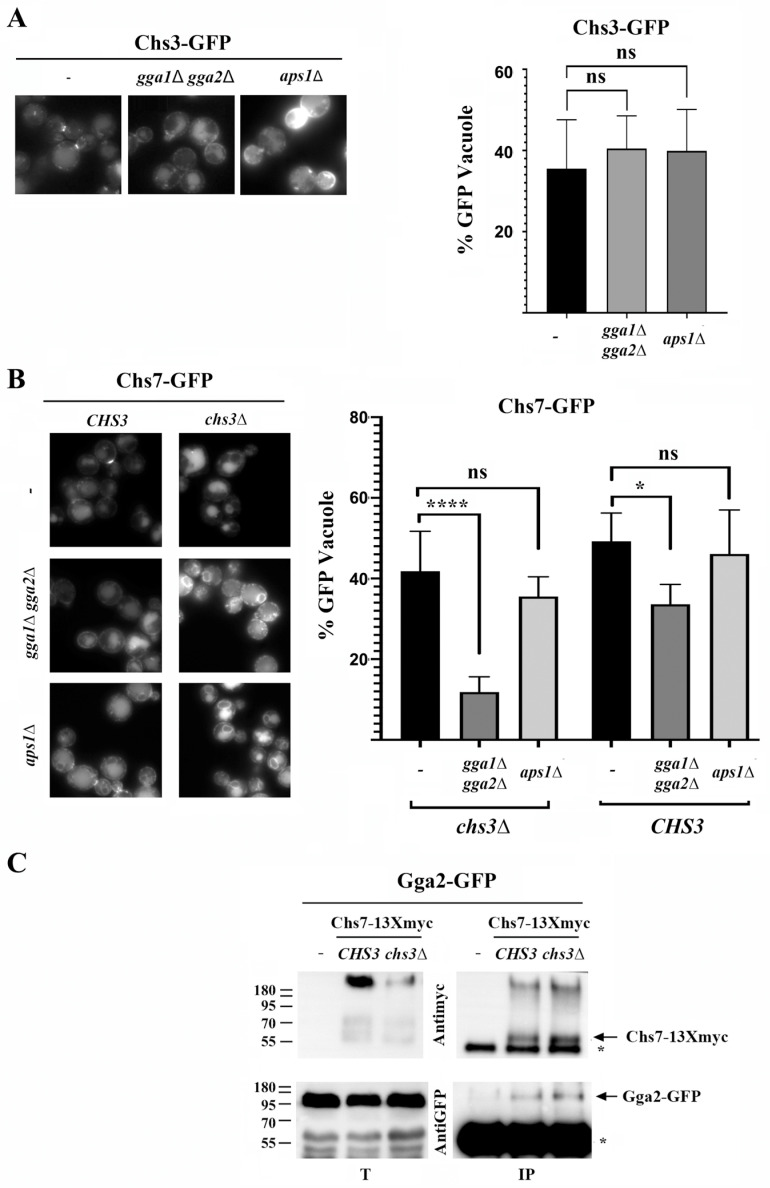
The role of the GGA complex in the sorting of CS3 components. (**A**) Left panels show the microscopic analysis of Chs3-GFP and right panels show the quantitative analysis of the relative amounts of the free-GFP band of Chs3-GFP in the indicated mutants. (**B**) Left panels show the microscopic analysis of Chs7-sfGFP and right panels show the quantitative analysis of the relative amounts of the free-sfGFP band associated with the vacuolar degradation of Chs7-sfGFP in the indicated mutants. The traffic is assessed in the presence or absence of Chs3 as indicated. (**C**) CoIP is between Chs7 and Gga2. Chs7-13xmyc is immunoprecipitated with anti-myc antibodies and the immunoprecipitate products (IPs) developed with anti-myc and anti-GFP antibodies. Note the formation of an immunoprecipitate between Chs7 and Gga2 independently of the presence of Chs3. Total extracts (T) are included as controls and the corresponding protein bands are marked with arrowheads. Bar plots represent the average value of at least 3 experiments (*n* = 3) and SD is indicated by the error bars in each case. Statistical significance is calculated as indicated in the MM. *p*-Values notation: ns = no significative; * (*p* < 0.05) and **** (*p* < 0.0001).

The anterograde transport of a functional Chs3/Chs7 complex to the PM depends on the exomer complex (Figure 1 and [33]). However, the sorting of Chs3 at the Golgi depends on the balance between this and its endosomal recycling mediated by the AP-1 and GGA adaptors [26,34]. Considering this, we analyzed how the individual sorting of these proteins at the TGN was achieved. In the absence of the AP-1 or GGA complexes, Chs3 is not efficiently recycled at the TGN and accumulates at the PM (Figure 5A, left panels); but its traffic to the vacuole seems to be maintained based on the intensity of the free-GFP band (Figure 5A, right panel). In agreement with this result, the traffic of orphan ^∆126^Chs3 to the vacuole was not dependent on the GGA complex (Figure S4B). In contrast, the traffic of orphan Chs7 to the vacuole was strongly reduced in the *gga1∆ gga2∆* mutant as determined by the significant reduction in the intensity of the free-GFP band and the vacuolar staining in this mutant (Figure 5B). Accordingly, Chs7 interacts physically with Gga2 based on CoIP experiments, and this interaction is independent of Chs3 (Figure 5C). However, its traffic to the vacuole in the absence of AP-1 was not significantly reduced. The penetrance of the phenotype associated with the absence of the GGA complex was significantly reduced in a wild-type strain (Figure 5B) when Chs7 formed a complex with the Chs3 protein. This indicated that most but not all of the Chs7 molecules followed Chs3 in its traffic from the TGN. These results indicated that the GGA complex should play a definitive role in the sorting of orphan Chs7 toward the vacuole while having an indirect role in the sorting of the Chs3 protein at the TGN, as previously reported [34].

**The traffic of Chs7 defines a general route for multiple proteins.** The results presented so far reveal a default route to the vacuole for unassembled proteins but also point to Chs7 as a protein that exceeds its previously described role as an ER chaperone, making it tempting to compare its trafficking to that of other proteins involved in ER sorting.

**Figure 6:**
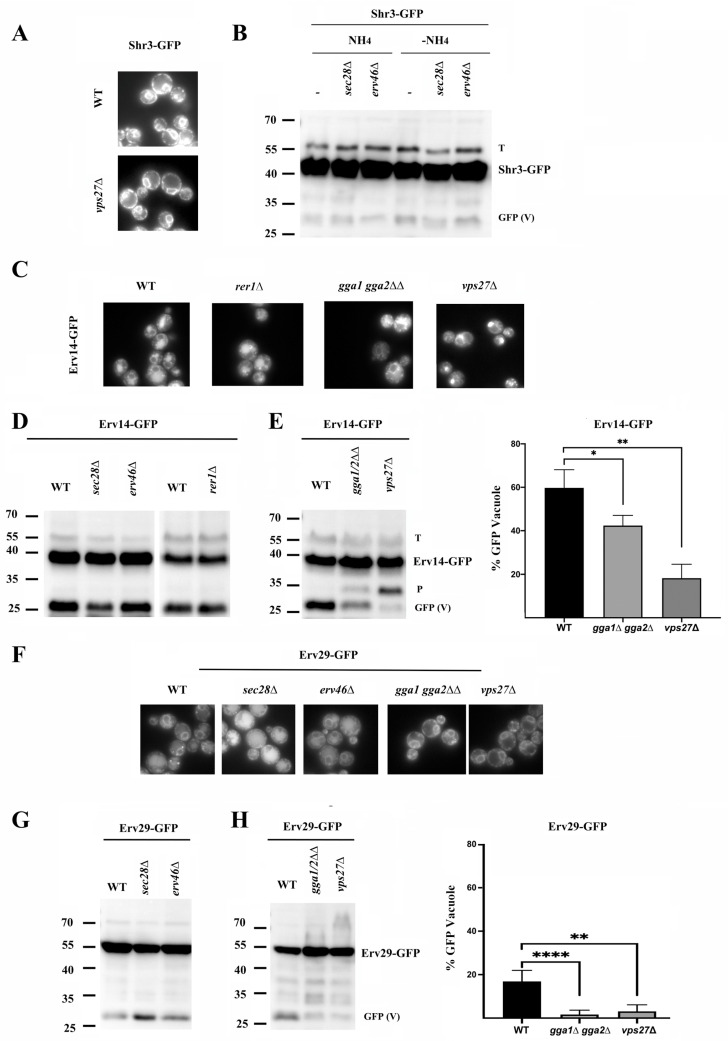
The traffic route of some ER adaptors. (**A**) The localization of Shr3-GFP in the indicated strains. (**B**) A Western blot of Shr3-GFP obtained from strains grown in the presence or absence of NH_4_. Note the minor intensity of the potential free-GFP bands. Tubulin (T) is used as the loading control. (**C**) The localization of Erv14-GFP in the indicated mutants. (**D**) A Western blot showing the levels of Erv14-GFP in the indicated COPI and COPI-adaptor mutants. Note the levels of the free-GFP band as indicative of vacuolar degradation. (**E**) A Western blot showing the levels of Erv14-GFP in the indicated mutants and quantitative analysis of the results. Note the appearance of the proteasomal degradation band. (**F**) The localization of Erv29-sfGFP in the indicated strains. (**G**) A Western blot showing the levels of Erv29-sfGFP in the indicated COPI or COPI-adaptor mutants. (**H**) A Western blot showing the levels of Erv29-sfGFP in the indicated mutants and the quantitative analysis of the results. Bar plots represent the average values of the relative levels of the free-GFP band from at least three independent experiments (*n* = 3). SD is indicated by the error bars and statistical significance is calculated with respect to the data from the control strain. GFP (V) refers to the free-GFP band and its intensity serves as a marker of vacuolar protein degradation. *p*-Values notation: * (*p* < 0.05); ** (*p* < 0.01) and **** (*p* < 0.0001).

Shr3, like Chs7, was originally described as a dedicated chaperone that prevents the inappropriate folding of Gap1 at the ER, allowing its loading into COPII vesicles [35]. However, Shr3 showed strict ER localization (Figure 6A). Also, we could not detect significant amounts of free-GFP regardless of the ammonium levels in the media (Figure 6B). Consequently, the absence of Vps27 did not induce Shr3 accumulation in endosome E (Figure 6A). Moreover, the deletion of COPI components did not result in free-GFP bands of increased intensity (Figure 6B). Therefore, Shr3 behaved as a typical ER protein, differing clearly from Chs7, with which it had been previously compared [16].

Most of the conventional ER sorting adaptors typically showed clear localization at the ER (Supplementary Figure S3). However, among them, Erv14 also showed vacuolar staining, a result that prompted us to directly test its traffic in different mutants. Erv14 was originally described as a typical ER protein that is loaded into COPII vesicles [36]. Therefore, it was proposed that it was recycled from the early Golgi back to the ER by COPI; however, Erv14 lacks COPI recognition motifs [37]. Our results clearly showed that Erv14 localized at the ER, but a significant part of this protein reached the vacuole as shown by the strong vacuolar staining observed and the appearance of the typical 27 kDa free-GFP band (Figure 6C,D). This traffic to the vacuole was absent in the Erv14^KS^ mutant (Figure S6B), which suggested that it was dependent on the export of the protein from the ER [36]. Moreover, vacuolar degradation was not increased in the absence of the COPI subunit Sec28 or the COPI adaptors Erv46 and Rer1 and was not reduced in the *apl6∆* or *vps10∆* mutants (Figure S6). However, Erv14 accumulated extensively at endosome E in the *vps27∆* mutant (Figure 6C). Additionally, its traffic to the vacuole was reduced in this mutant, as shown by a reduction in the free-GFP band (Figure 6E). Moreover, its traffic to the vacuole was also partially reduced in the *gga1∆ gga2∆* mutant (Figure 6C,E). Interestingly, blocking its traffic to the vacuole caused the appearance of a degradation band (Figure 6E, band P) compatible with proteasomal degradation.

Along the same lines, the ER adaptor Erv29 also partially trafficked to the vacuole, but in this case, this traffic was increased in the absence of the COPI component Sec28 (Figure 6F,G), in accordance with the presence of a COPI binding retrieval motif in the C-terminal domain of the protein [38]. Moreover, the traffic of Erv29 to the vacuole was reduced in the absence of GGA and Vps27 based on microscopic and Western blot analyses (Figure 6F,H). However, a significant accumulation of Erv29 at endosome E was not detected in the *vps27∆* mutant. Taken together, the results obtained suggest that Chs7, Erv14, and Erv29 follow a similar traffic route to the vacuole, differing essentially in their recycling by COPI. The absence of the COPI trafficking for Erv14 may result in a more direct traffic of this protein to the vacuole.

**Figure 7:**
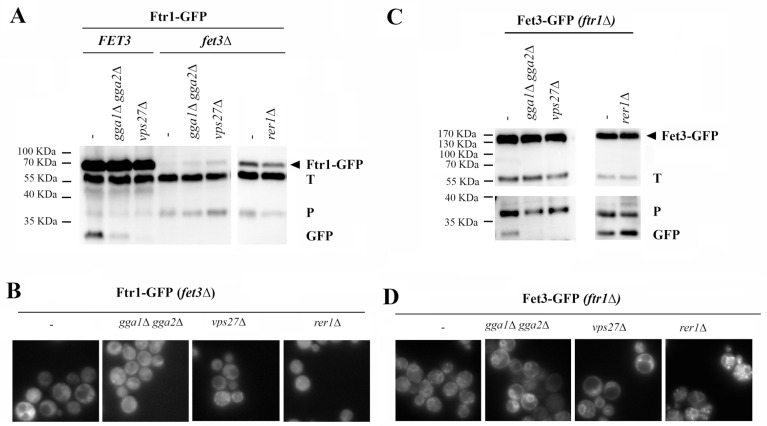
The traffic route of the components of the Fet3/Ftr1 complex. (**A**) A Western blot of Ftr1-GFP in the indicated mutants in the presence or absence of Fet3 as indicated. Note the complete disappearance of the free-GFP band and the appearance of a band indicative of incomplete proteasomal degradation (P) in the *fet3∆* mutant (**B**) The localization of Ftr1-GFP in the absence of Fet3 in the indicated mutants. (**C**) A Western blot of Fet3-sfGFP in the indicated mutants in the absence of Ftr1. The lower part of the gel is overexposed to show details. (**D**) The localization of Fet3-sfGFP in the indicated mutants in the absence of Ftr1. Tubulin (T) was used as loading control in Western blot experiments. P indicates a band associated with incomplete proteasomal degradation. GFP (V) refers to the free-GFP band and its intensity serves as a marker of vacuolar protein degradation.

However, Chs7 is shown to form a true complex with Chs3 (Figure 1, [19]); therefore, its traffic can also be compared with that of proteins that form complexes at the ER. Among these, the high-affinity iron transporter formed by Fet3 and Ftr1 proteins has been shown to assemble in the ER [39,40]. The correctly assembled complex, visualized through the tagging of Ftr1, efficiently reached the PM from where it is eventually trafficked to the vacuole, as determined by the appearance of the free-GFP band (Figure 7A). In agreement with classical endocytic traffic, its traffic to the vacuole was reduced in the absence of the GGA complex and prevented in the *vps27∆* mutant, as determined by the amounts of the free-GFP band. Neither Ftr1 nor Fet3 proteins can reach the PM individually. Therefore, their traffic depends entirely on the formation of a protein complex at the ER [39,40]. Based on this, we analyzed the individual traffic of each component of the complex. In the absence of Fet3, the total levels of Ftr1-GFP were significantly reduced (Figure 7A) and the protein accumulated partially at the ER. Nonetheless, intense cytosolic staining was detected but, as expected, not along the PM (Figure 7B). Interestingly, no free-GFP band was observed (Figure 7A) and the vacuole remained unstained (Figure 7B). Furthermore, a significant band of approximately 37 kDa was detected in the Western blot analysis that had been previously associated with incomplete proteasomal degradation (P) [41]. The further removal of Ggas, Vps27, or Rer1 did not significantly change the distribution of the protein based on the Western blot and microscopic analysis, but the elimination of the COPI component Rer1 reduced the ER localization of Ftr1. Altogether, these results indicate that in the absence of Fet3, Ftr1 traffic is blocked at the ER, and the protein is probably degraded by the proteasome based on the cytosolic distribution of the GFP signal, which is consistent with the low levels of Ftr1-GFP and the appearance of the 37kDa processing band [41]. The traffic of Fet3 to the PM was also prevented in the *ftr1∆* mutant showing a strong ER signal (Figure 7D). However, we could detect significant amounts of the free-GFP vacuolar band (Figure 7C). These amounts were increased in the COPI mutant *rer1∆* (see the right panel of Figure 7C) as expected due to the role that has been described for this adaptor in the retrograde traffic of unassembled Fet3 [39]. In contrast, this band disappeared in the *gga1∆ gga2∆* and *vps27∆* mutants and, accordingly, the vacuole was essentially free of staining in the *vps27∆* mutant in which the accumulation of the protein was detected at endosome E. Thus, unlike Ftr1, the retention of Fet3 at the ER is promoted by the COPI-mediated retrograde transport as described in [39]. However, Fet3 is not fully degraded by the proteasome and an eventual accumulation of the protein is resolved through its traffic to the vacuole via the endosomal compartment.

## 4. Discussion

Chs3 has been used as a model for the study of the intracellular traffic of proteins and, consequently, its study has led to the discovery of many dedicated proteins required for its transport, including the exomer complex involved in the sorting of proteins at the TGN in most fungi [14,42]. However, recently, it has been proposed that Chs7, originally described as an ER chaperone, forms a complex with Chs3 at the ER that travels along the secretory route [19]. Our work confirms this initial proposal and shows that the Chs3/Chs7 complex traffics from the ER to the PM. Afterward, this complex is endocytosed and most of it is recycled at the TGN, with a small amount reaching the vacuole for its degradation (Figure S1).

**Unassembled components of chitin synthase are exported from the ER.** We do not yet know how the formation of the functional Chs3/Chs7 complex affects the different steps in the trafficking of chitin synthase. Nevertheless, the work in this study on the individual traffic of both components led us to the original observation that Chs7 reaches the vacuole even in the absence of Chs3 (Figure 1). Since Chs3 requires Chs7 for exiting the ER, it was not detected in the vacuolar compartment of the *chs7∆* mutant. However, unexpectedly, the truncated protein ^∆126^Chs3 individually reached the vacuole for its degradation in the absence of Chs7, providing a model for studies on the individual traffic of both components of the CS3 complex. Our results dismiss the involvement of autophagy in the traffic of these orphan proteins and show that they exit the ER in COPII vesicles.

Both Chs7 and Chs3 can interact directly with the Erv14 adaptor, facilitating the loading of the Chs3/Chs7 complex into COPII vesicles following the canonical route for the export of integral transmembrane proteins [37]. However, while orphan Chs7 can be loaded individually into these vesicles, the extensive aggregation of Chs3 in the absence of Chs7 [16,17] prevents its loading despite its interaction with Erv14. ^∆126^Chs3 interacted poorly with Erv14, but the formation of the Chs7/^∆126^Chs3 complex facilitated its export from the ER in COPII vesicles. Moreover, ^∆126^Chs3 lacks its oligomerization domain, which prevents its aggregation in the absence of Chs7 (Figure 2E), and all the other Chs3 proteins lacking the oligomerization domain can also exit the ER in the absence of Chs7 (Figure S2). This suggests that the oligomerization of the protein is what prevents its loading into COPII vesicles independently of its potential interaction with the cargo adaptor Erv14.

We cannot be sure about the existence of additional adaptors facilitating the ^∆126^Chs3 loading into COPII vesicles, but this loading would likely occur through the ill-defined bulk flow mechanism. This unspecific loading system seems to be less efficient than the one mediated by Erv14 since the amount of this protein reaching the vacuole was consistently reduced in the absence of Chs7 (compare the intensity of the GFP band in Figure 1D, Figure 3D and Figure 4D). This result is in agreement with previous reports showing a reduced export rate for different proteins in the absence of their corresponding ER adaptors (reviewed in [30]).

**Unassembled CS components are recycled before being targeted to the vacuole.** The functional Chs3/Chs7 complex apparently passes freely through the early Golgi without being recognized by COPI. However, both orphan Chs7 and ^∆126^Chs3 were efficiently recognized by COPI and returned to the ER upon their arrival at the Golgi (Figure 3). This retrograde transport would be fully consistent with a QC mechanism that facilitates the recovery of unassembled protein components of complexes from the Golgi to promote their refolding in the ER [39,43].

It is quite likely that the reduced interaction of Chs7 with ^∆126^Chs3 would facilitate the arrival of orphan molecules to the Golgi, which would explain the COPI-mediated recycling of functional Chs7/^∆126^Chs3 complexes [17]. However, the recognition of Chs7/^∆126^Chs3 complexes is achieved through a C-terminal di-lysine motive on ^∆126^Chs3 [17], which seems to be inactive for orphan ^∆126^Chs3 (Figure 3D). Therefore, the Chs7^/∆126^Chs3 complex and the orphan proteins may be recognized through different mechanisms. This possibility is consistent with our results showing a rather different penetrance level in the COPI mutants depending on the cargo and suggests the coexistence of multiple mechanisms for protein recognition by COPI, findings that are in agreement with the prevalent model [30].

**Unassembled CS components are sorted at the TGN for delivery to the vacuole.** The continuous traffic of the orphan CS components to the vacuole indicates that COPI recycling cannot cope with the total amount of synthesized proteins. As a consequence, some of the orphan proteins follow their route through the Golgi and reach the vacuole without passing through the PM beforehand. Therefore, we examined how these individual proteins get sorted at the TGN for their delivery to the vacuole. Our results indicated that this traffic was not mediated by the classical vacuolar pathways CPY (Vps10 receptor) or AP-3 but instead relied on ESCRT machinery. Thus, these proteins needed to be recruited at the TGN and directed to the endosomal system where they would ultimately be recognized by ESCRT and processed. According to our results, Chs7 directly interacts with the protein adaptor Gga2 and its traffic to the vacuole is clearly dependent on the GGA complex (Figure 5), indicating that the GGA complex directly participates in the recruitment of Chs7 for its traffic to the vacuole. However, we do not know how this interaction takes place because Chs7 lacks a canonical acidic di-leucine domain. Also, it is not predicted to be ubiquitinated based on its secondary structure; therefore, it lacks the conventional recognition motifs for the GGA complex [44]. Interestingly, ^∆126^Chs3 traffic to the vacuole was found to be independent of this complex (Figure S4), which is similar to what has been proposed for the wild-type Chs3 protein [45]. Thus, the different involvement of the GGA complex in the traffic of Chs7 and Chs3 supports the differential role of GGA proteins in the sorting of different TM proteins into the vacuole [45]. Surprisingly, in wild-type cells, the traffic of Chs7 to the vacuole after binding to Chs3 remains partially dependent on GGA, which suggests that orphan Chs7 molecules are present at the TGN. However, whether these unassembled molecules arrive directly from the ER or are generated from functional Chs3/Chs7 complexes remains unknown.

We then set out to determine the behavior of these proteins when their traffic to the vacuole is blocked. We detected an intense accumulation of these proteins at endosome E in the *vps27∆* mutant, suggesting that this could be the major final destination for these proteins. However, when the traffic of orphan Chs7 to the vacuole was reduced in the *gga1∆ gga2∆* mutant, we observed a significant increase in the ER signal (compare ER signals in Figure 5B). This accumulation was reduced in double mutants devoid of different COPI components, in which the intensity of the free-GFP band was increased compared to that of the single *gga1∆ gga2∆* mutant (Figure S5). The results, when taken together, suggest that the portion of Chs7 that is not targeted to the vacuole could be recycled back to the ER by COPI from the late Golgi compartment, as has been previously proposed for other proteins [46]. In the case of ^∆126^Chs3, we detected a significant increase in the processing band associated with proteasomal degradation in the *vps27∆* mutant. This degradation is unlikely to be mediated by endosome and Golgi-associated degradation (EGAD) [47] because the traffic of orphan ^∆126^Chs3 was not affected in the *tul1∆* mutant (Figure S4). This suggests that part of this protein is recycled back to the ER, where it could be a substrate for ERAD/INMAD, as recently described for the wild-type protein [18].

In summary, our results show how yeast cells monitor the formation of a protein complex in the ER, guaranteeing that the individual components that are not properly assembled can be recycled back to the ER from the early Golgi for new opportunities for assembling. In addition, we also show how a potential excess of unassembled proteins is sorted at the TGN for its delivery to the vacuole through the multivesicular body route.

**Chitin synthase trafficking reflects general rules for protein homeostasis in yeast cells.** The original proposal of Chs7 as a resident ER chaperone led us to compare its traffic to that of other molecules of the same type as Shr3. However, our results clearly indicated that there are significant differences in the trafficking of these two proteins, with Shr3 behaving rather strictly as an ER resident chaperone, as reported [35], which limits its biological function to the ER. Surprisingly, the traffic of Chs7 was much more similar to that of some of the classical ER adaptors, such as Erv29 and Erv14, which were also able to reach the vacuole through the MVB pathway. However, while a potential excess of Erv14 is eliminated essentially through its delivery to the vacuole, Erv29 is first recycled through COPI, as previously reported [37], and only the non-recycled part of the protein is evacuated to the vacuole. Proteasomal degradation clearly contributes to the homeostasis of Erv14 if the traffic to the vacuole is blocked (Figure 6E), but its role in the homeostasis of Erv29 is unclear. Interestingly both proteins, similar to Chs7, rely on the GGA complex for their traffic to the vacuole. This suggests that this complex is directly involved in the clearance of proteins incorrectly arriving at the Golgi.

There are also significant similarities between the traffic of the chitin synthase complex and the high-affinity iron transporter formed by Fet3 and Ftr1. Both complexes follow the secretory route to the PM and are later endocytosed to be degraded in the vacuole through the MVB [27,39]. However, the traffic of the individual components of the high-affinity iron transporter differs from that of the CS complex. The unassembled iron permease Ftr1 accumulated at the ER is rapidly degraded, likely by the proteasome, and does not reach the vacuole, clearly differing from the behavior of both Chs7 and Chs3. In contrast, the unassembled ferro-oxidoreductase Fet3 is partially recycled by COPI, although it eventually reaches the vacuole through the MVB pathway. Its accumulation was also partially relieved by the proteasome, strongly resembling the behavior of the unassembled ^∆126^Chs3. Thus, our results support that, in addition to the role of ERAD in the degradation of unassembled components of protein complexes [48], vacuolar degradation via ESCRT also plays a significant role in the maintenance of the balance between the different components of protein complexes.

**Figure 8:**
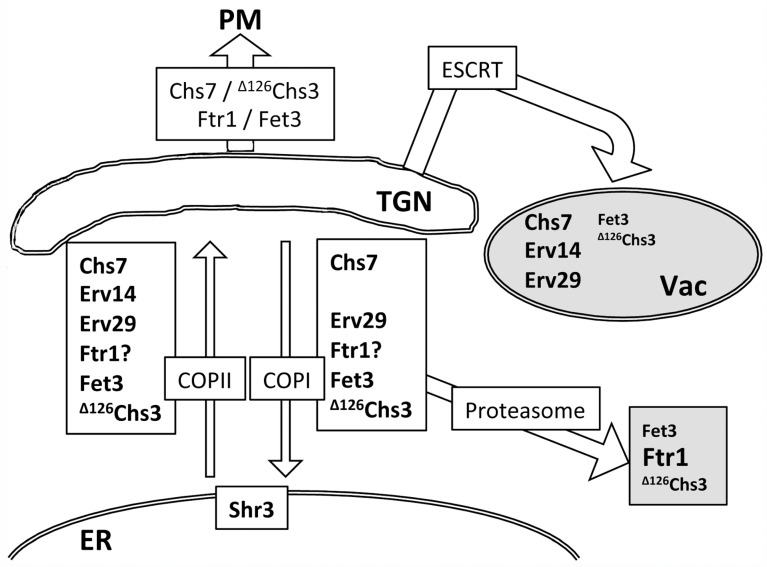
A schematic representation of the traffic of different components of protein complexes and ER adaptors in budding yeast. Unassembled components of protein complexes and ER adaptors can be recycled from the Golgi by the COPI machinery. However, while the eventual excess of some of them is removed through proteasomal degradation, others are degraded in the vacuole via the ESCRT complex. Some unassembled subunits may undergo both proteasomal and vacuolar degradation. Comparative degradation levels are indicated by the size of the letters, with larger letters representing components with higher levels of degradation.

A comparative Western blot analysis of these proteins (Figure S7) appears to reflect their trafficking patterns The results of this analysis are integrated in Figure 8. The strong intensity of the free-GFP band detected for Chs7 and Erv14 proteins indicates their extensive trafficking to the vacuole, which correlates with their pronounced accumulation at endosome E in the *vps27∆* mutant (Figure 8). In contrast, the reduced intensity of free-GFP bands for Erv29 and Fet3 suggests the efficient recycling of these proteins by COPI. The appearance of the proteasomal degradation band (P) denotes significant proteasomal degradation most likely via ERAD, which, in the case of Ftr1, led to reduced levels of the total protein. To date, it is unclear if this protein can exit the ER individually [40,49] (Figure 7B). Our results also indicate that an excess of the Fpr2 protein, an ER peptidyl-prolyl cis-trans isomerase (PPIase), is targeted to the vacuole despite the fact that it has been recently described as a cargo of the Erv41/Erv46 adaptor complex [31]. Our data highlight Chs7, an integral TM protein, as a new cargo of the Erv41/Erv46 complex. Unlike Chs7, Fpr2 and other described cargoes of this complex are soluble proteins, indicating that the Erv41/Erv46 complex can recruit proteins through different mechanisms.

A distinct combination of COPI recycling, proteasomal, and vacuolar degradation enables yeast cells to maintain protein homeostasis at the ER by differentially handling unassembled subunits, providing new opportunities for refolding/reassembling before they are ultimately degraded.

## Data Availability

The original contributions presented in the study are included in the article/supplementary material, further inquiries can be directed to the corresponding author.

## Supplementary Materials

The following supporting information can be downloaded at: https://www.mdpi.com/article/10.3390/jof11030221/s1, Figure S1: Colocalization of Chs3-GFP and Chs7-mCherry along the secretory pathway. Both proteins were tagged in the chromosome and their localization assessed by fluorescence microscopy; Chs3 was observed in the GFP channel and Chs7 in the mCherry channel. Note that their localization depend on the mutant genetic background. Also, note the almost perfect colocalization of both signals, but how the overall accumulation of m-Cherry in the vacuole is stronger; Figure S2: (A) Localization of the indicated versions of Chs3 in the presence or absence of Chs7 as indicated. Note the ER retention of all constructs in the *chs7∆* mutant except in the case of ^∆126^Chs3. (B) Localization of the indicated versions of Chs3 in the absence of Chs7. Note the strong vacuolar signal in both strains; Figure S3: Localization of the indicated proteins. All images were obtained from SGD except for Chs7-GFP (*chs3∆*), which is from our lab; Figure S4: The individual traffic of ^∆126^Chs3 to the vacuole; Figure S5: Relative levels of free-GFP bands from Chs7-GFP in the absence of Chs3 in the indicated mutants. Note the partial rescue of vacuolar degradation in the *gga1∆ gga2∆* mutant caused by the deletion of different COPI proteins; Figure S6: (A) Western blot of the Erv14-GFP protein in the indicated mutants. Note the strong signal of the free-GFP band that is strongly reduced in the *vps27∆* mutant and moderately in the *gga1∆ gga2∆* mutant. Blockage of the traffic to the vacuole in the ESCRT-0 mutant *vps27∆* facilitates the proteasomal degradation of the protein as deduced from the more intense proteasomal band (P) detected in this mutant. (B) Western blot of the Erv14-GFP protein and its non-functional version, Erv14^KS^-GFP (Powers and Barlowe, 2002) in the indicated mutants and the wild type strain (-). Note how Erv14^KS^-GFP is virtually absent from the vacuole in the wild type and in the COPI or COPI-adaptor mutants. GFP (V) refers to the free-GFP band and its intensity serves as a marker of vacuolar protein degradation; Figure S7: Comparative analysis of the traffic of the individual components of different protein complexes and ER resident proteins; Table S1: Strains used in this work; Table S2: Plasmids used in this work.
