# Supplementary material for: Different Proteostasis Mechanisms Facilitate the Assembly of Individual Components on the Chitin Synthase 3 Complex at the Endoplasmic Reticulum
Source: J Fungi (Basel). 2025 Mar 14;11(3):221. doi: 10.3390/jof11030221 (PMC11943272; doi:10.3390/jof11030221)
Supplement: Supplementary file 1 [file jof-11-00221-s001.zip › jof-3490392-supplementary tables.pdf]

Table S1. Strains used in this work.

Strain	Genotype	Origin/Reference
**CRM67**	W303, mat a (*leu2-3,112 trp1-1 can1-100 ura3-1 ade2-1 his3-11,15*)	Lab collection
**CRM659**	BY4741; Mat a; (*his3Δ1 leu2Δ0 met15Δ0 ura3Δ0*)	Lab collection
**CRM1590**	W303, mat a, *chs3*Δ::*natMx4*	Lab collection
**CRM101**	W303, mat α, *chs3*Δ::*URA3*	Lab collection
**CRM1348**	W303, mat α, *chs3*Δ::*URA3 chs7*Δ::*natMx4*	Lab collection
**CRM3898**	W303; Mat a; Chs7-sfGFP*::*kanMx4*	This work
**CRM3952**	W303; Mat a; *chs3*Δ::*natMx4* Chs7-sfGFP::*kanMx4*	This work
**CRM4665**	W303; Mat a; Chs7-sfGFP::*kanMx4 pep4*Δ: *natMx4 chs3*Δ::*hphNT1*	This work
**CRM1278**	W303; Mat α; *chs3*Δ::*URA3 chs5*Δ::*natMx4*	Lab collection
**CRM4474**	W303; Mat α; *chs3*Δ::*URA3 chs5*Δ::*natMx4 chs7*Δ::*HIS3*	This work
**CRM4785**	W303; Mat α; *chs3*Δ::*URA3 chs5*Δ::*natMx4* Chs7-sfGFP::*HIS3*	This work
**CRM4300**	W303; Mat a; *chs3*Δ::*natMx4* Chs7-sfGFP::*kanMx4 atg1*Δ::*hphNT1*	This work
**CRM4617**	W303; Mat a; *chs3*Δ::*natMx4 atg1*Δ::*hphNT1 chs7*Δ::*HIS3*	This work
**CRM4638**	*sec23*(ts); Mat α; *chs3*Δ::*kanMx4; (ura3 leu2)*	This work
**CRM4685**	*sec23*(ts); Mat α; *chs3*Δ::*hphNT1* Chs7-sfGFP::*kanMx4*	This work
**CRM4649**	*sec23*(ts); Mat α; *chs3*Δ::*kanMx4 chs7*Δ::*natMx4*	This work
**CRM4559**	*sec31-1*(ts); Mat α; *chs3*Δ::*LEU2 chs7*Δ::*HIS3; (ura3 leu2 trp1 his3 met15)*	This work
**CRM1670**	*sec31-1*(ts); Mat α; *chs3*Δ::*LEU2*	Muñiz M.
**CRM4592**	*sec31-1*(ts); Mat α; *chs3*Δ::*LEU2* Chs7-sfGFP::*kanMx4*	This work
**CRM4667**	*sec31-1*(ts); Mat α; *chs3*Δ::*hphNT1*	This work
**CRM4738**	W303; Mat a; *chs3*Δ::*LEU2 erv14*Δ::*kanMx4* Chs7-sfGFP::*URA3*	This work
**CRM4556**	W303; Mat α; *chs3*Δ::*URA3 erv14*Δ::*kanMx4 chs7*Δ::*HIS3*	This work

**CRM4778**	Erv14-mCi::*HIS3* (*ura3 leu2 trp1 met15*)	Muñiz M.
**CRM4848**	Erv14-mCi::*HIS3 chs3*Δ::*URA3*	This work
**CRM4856**	Erv14-mCi::*HIS3 chs3*Δ::*URA3 chs7*Δ::*kanMx4*	This work
**CRM4735**	W303; Mat a; *chs3*Δ::*natMx4 sec28*Δ::*kanMx4* Chs7-sfGFP::*URA3*	This work
**CRM4736**	W303; Mat a; *chs3*Δ::*LEU2 rer1*Δ::*kanMx4* Chs7-sfGFP::*URA3*	This work
**CRM4676**	W303; Mat a; *chs3*Δ::*natMx4* Chs7-sfGFP::*kanMx4 erv46*Δ::*hphNT1*	This work
**CRM4554**	W303; Mat a; *chs3*Δ::*natMx4 sec28*Δ::*kanMx4 chs7*Δ::*HIS3*	This work
**CRM4390**	*chs3Δ::LEU2 rer1*Δ::*kanMx6 chs7*Δ::*hph; (ade2 trp1 ura3 leu2 his3)*	Muñiz M.
**CRM4733**	W303; Mat a; *chs3*Δ::*natMx4 erv46*Δ::*hphNT1 chs7*Δ::*HIS3*	This work
**CRM4413**	W303; Mat a; *chs3*Δ::*natMx4 CHS7*-VC::*HIS3*	This work
**CRM4680**	W303; Mat a; *chs3*Δ::*natMx4 CHS7*-VC::*HIS3 SEC28*-VN::*kanMx4*	This work
**CRM4681**	W303; Mat a; *chs3*Δ::*natMx4 CHS7*-VC::*HIS3 ANP1*-VN::*kanMx4*	This work
**CRM4298**	W303; Mat a; *chs3*Δ::*natMx4* Chs7-sfGFP::*kanMx4 apl6*Δ::*hphNT1*	This work
**CRM4218**	W303; Mat α; *chs3*Δ::*URA3 chs7*Δ::*natMx4 apl6*Δ::*hphNT1*	This work
**CRM4295**	W303; Mat α; *chs3*Δ::*URA3 chs7*Δ::*natMx4 apl6*Δ::*hphNT1 atg1*Δ::*kanMx4*	This work
**CRM4673**	W303; Mat a; *chs3*Δ::*natMx4* Chs7-sfGFP::*kanMx4 vps10*Δ::*hphNT1*	This work
**CRM4767**	W303; Mat α; *chs3*Δ::*URA3 chs7*Δ::*natMx4 vps10*Δ::*hphNT1*	This work
**CRM4821**	W303; Mat a; *chs3*Δ::*natMx4* Chs7-sfGFP::*kanMx4 apl6*Δ::*hphNT1 vps10*Δ::*URA3*	This work
**CRM4836**	W303; Mat α; *chs3*Δ::*URA3 chs7*Δ::*natMx4 vps10*Δ::*hphNT1 apl6*Δ::*kanMx4*	This work
**CRM4374**	W303; Mat a; *chs3*Δ::*NAT* Chs7-sfGFP::*kanMx4 vps27*Δ::*hphNT1*	This work
**CRM4228**	W303; Mat α; *chs3*Δ::*URA3 chs7*Δ::*natMx4 vps27*Δ::*kanMx4*	This work
**CRM1951**	W303; Mat α; *chs3*Δ::*URA3 vps27*Δ::*natMx4*	Lab collection

**CRM4604**	W303; Mat a; *gga2*Δ::*hphNT1 gga1*Δ::*natMx4 chs3*Δ::*kanMx4*	Lab collection
**CRM1777**	W303; Mat α; *chs3*Δ::*URA3 aps1*Δ::*kanMx4*	Lab collection
**CRM4705**	W303; Mat a; *gga2Δ::hphNT1 gga1*Δ::*natMx4 chs3*Δ::*kanMx4* Chs7-sfGFP::*URA3*	This work
**CRM4763**	W303; Mat α; *chs3*Δ::*URA3 aps1*Δ::*kanMx4* Chs5-mCherry::*natMx4* Chs7-sfGFP::*HIS3*	This work
**CRM4975**	BY4741; Mat a; Gga2-GFP::*hphNT1* Chs7-13xMyc::*kanMx6 chs3*Δ::*URA3*	This work
**CRM4864**	W303; Mat a; Shr3-GFP::*hphNT1*	This work
**CRM4915**	W303; Mat a; Shr3-GFP::*hphNT1 vps27*Δ::*kanMx4*	This work
**CRM4926**	W303; Mat a; Shr3-GFP::*hphNT1 erv46*Δ::*kanMx4*	This work
**CRM4927**	W303; Mat a; Shr3-GFP::*hphNT1 sec28*Δ::*kanMx4*	This work
**CRM3621**	W303; Mat a; *gga2*Δ::*hphNT1 gga1*Δ::*natMx4*	Lab collection
**CRM1565**	BY4741; Mat a; *rer1*Δ::*kanMx4*	Lab collection
**CRM3382**	W303; Mat a; *vps27*Δ::*kanMx4*	Lab collection
**CRM4925**	W303; Mat a; Erv29-sfGFP::*URA3*	This work
**CRM4942**	W303; Mat a; Erv29-sfGFP::*URA3 sec28*Δ::*hphNT1*	This work
**CRM4950**	W303; Mat a; Erv29-sfGFP::*URA3 erv46*Δ::*hphNT1*	This work
**CRM4960**	W303; Mat a; Erv29-sfGFP::*URA3 vps27*Δ::*hphNT1*	This work
**CRM4961**	W303; Mat a; Erv29-sfGFP::*URA3 gga2*Δ::*kanMx4 gga1*Δ*::natMx4*	This work
**CRM3635**	BY4741; Mat a; Ftr1-GFP::*kanMx4*	Lab collection
**CRM3695**	BY4741; Mat a; Ftr1-GFP::*kanMx4 gga2*Δ::*hphNT1 gga1*Δ::*NAT*	Lab collection
**CRM4952**	BY4741; Mat a; Ftr1-GFP::*kanMx4 vps27*Δ::*hphNT1*	This work
**CRM4953**	BY4741; Mat a; Ftr1-GFP::*kanMx4 fet3*Δ::*URA3*	This work
**CRM4959**	BY4741; Mat a; Ftr1-GFP::*kanMx4 fet3*Δ::*URA3 gga2*Δ::*hphNT1 gga1*Δ::*natMx4*	This work

**CRM4974**	BY4741; Mat a; Ftr1-GFP::*kanMx4 fet3*Δ::*URA3 vps27*Δ::*hphNT1*	This work
**CRM4988**	BY4741; Mat a; Ftr1-GFP::*kanMx4 fet3*Δ::*URA3 rer1*Δ::*HIS3*	This work
**CRM4930**	W303; Mat a; Fet3-sfGFP::*HIS5 ftr1*Δ::*hphNT1*	This work
**CRM4946**	W303; Mat a; Fet3-sfGFP::*HIS5 ftr1*Δ::*hphNT1 vps27*Δ::*kanMx4*	This work
**CRM4967**	W303; Mat a; Fet3-sfGFP::*HIS5 ftr1*Δ::*hphNT1 gga2*Δ::*kanMx4 gga1*Δ::*natMx4*	This work
**CRM4985**	W303; Mat a; Fet3-sfGFP::*HIS5 ftr1*Δ::*hphNT1 rer1*Δ::*URA3*	This work
**CRM4935**	W303; Mat a; Fpr2-sfGFP::*HIS5*	This work
**CRM4803**	W303; Mat α; Chs7-mCherry::*natMx4 chs3*Δ::*URA3*	This work
**CRM4813**	W303; Mat α; Chs7-mCherry:: *natMx4 chs3*Δ::*URA3 chs5*Δ::*kanMx4*	This work
**CRM4820**	W303; Mat α; Chs7-mCherry:: *natMx4 chs3*Δ::*URA3 aps1*Δ::*kanMx4 chs5*Δ::*hphNT1*	This work
**CRM4824**	W303; Mat α; Chs7-mCherry:: *natMx4 chs3*Δ::*URA3 vps27*Δ::*hphNT1*	This work
**CRM4818**	W303; Mat α; Chs7-mCherry::*natMx4 chs3*Δ::*URA3 art4*Δ::*kanMx4*	This work
**CRM4366**	W303; Mat α; *chs3*Δ::*URA3 chs7*Δ::*natMx4 vam3*Δ::*kanMx4*	This work
**CRM4363**	W303; Mat α; *chs3*Δ::*URA3 chs7*Δ::*natMx4 vac8*Δ::*kanMx4*	This work
**CRM4349**	W303; Mat α; *chs3*Δ::*URA3 chs7*Δ::*natMx4 ire1*Δ::*kanMx4*	This work
**CRM4319**	W303; Mat α; *chs3*Δ::*URA3 chs7*Δ::*natMx4 vps21*Δ::*kanMx4*	This work
**CRM4313**	W303; Mat α; *chs3*Δ::*URA3 chs7*Δ::*natMx4 atg40*Δ::*kanMx4*	This work
**CRM4368**	W303; Mat α; *chs3*Δ::*URA3 chs7*Δ::*natMx4 lst1*Δ::*hphNT1*	This work
**CRM4784**	W303; Mat α; *chs3*Δ::*URA3 chs7*Δ::*HIS3 ubp3*Δ::*hphNT1*	This work
**CRM4933**	W303; Mat a; *gga2*Δ::*hphNT1 gga1*Δ::*natMx4 chs3*Δ::*kanMx4* Chs7-sfGFP::*URA3 sec28*Δ::*LEU2*	This work
**CRM4934**	W303; Mat a; *gga2*Δ::*hphNT1 gga1*Δ::*natMx4 chs3*Δ::*kanMx4* Chs7-sfGFP::*URA3 erv46*Δ::*LEU2*	This work
**CRM2971**	BY4742; Mat α; *gga1*Δ::*natMx4 gga2*Δ::*hphNT1*	Lab collection

**CRM1857**	BY4741; Mat a; *vps27*Δ::*kanMx4*	Lab collection
**CRM3135**	BY4741; Mat a; *atg1*Δ::*kanMx4*	Lab collection
**CRM2207**	BY4741; Mat a; *apl6*Δ::*kanMx4*	Lab collection

*sfGFP references to superfolderGFP.

Table S2. Plasmids used in this work.

Plasmid	Name	Origin/Reference
**CRM3371**	pFA6a-*hphNT1*	(Goldstein and McCusker, 1999)
**CRM2546**	pAG25 (*natMx4*)	(Goldstein and McCusker, 1999)
**CRM1188**	pUG6 (*kanMx4*)	(Goldstein and McCusker, 1999)
**CRM1191**	pUG72 (*URA3*)	Spang A.
**CRM4400**	pRS303(*HIS3*)	Segurado M.
**CRM1808**	pFA6a-*13xMyc::kanMx6*	Sánchez Y.
**CRM4682**	pFA6a-link-yo-sfGFP*(HIS5)*	Add gene
**CRM4683**	pFA6a-link-yo-sfGFP*(URA3)*	Add gene
**CRM3858**	pFA6a-sfGFP::*kanMx4*	(Pédelacq et al., 2006)
**CRM2653**	pFN21 (mCherry::*natMx4*)	(Sato et al., 2005)
**CRM3472**	pFA6a-**V**enus**N**terminal::*kanMx4*	(Sung and Huh, 2007)
**CRM3469**	pFA6a-**V**enus**C**terminal::*HIS3*	(Sung and Huh, 2007)
**CRM1994**	pFA6a-GFP::*kanMx4*	(Sato et al., 2005)
**CRM1995**	pFA6a-GFP::*hphNT1*	(Sato et al., 2005)
**CRM1131**	pRS315::*CHS3*-GFP	(Sacristan et al., 2013)
**CRM1130**	pRS315::*CHS3*	(Sacristan et al., 2013)
**CRM1256**	pRS315::$^{\Delta126}$*CHS3*-GFP	(Sacristan et al., 2013)
**CRM1270**	pRS313::$^{\Delta126}$*CHS3*-GFP	Lab collection
**CRM1299**	pRS314::$^{\Delta126}$*CHS3*-GFP	Lab collection
**CRM1084**	pRS315::*CHS3*-3xHA	(Sacristan et al., 2013)
**CRM1290**	pRS315::$^{\Delta126}$*CHS3*-3xHA	(Sacristan et al., 2013)
**CRM1721**	pRS315::$^{\Delta126}$*CHS3* $^{K1125/1126R}$-GFP ($^{\Delta126}$Chs3^{KK})	(Sacristan et al., 2013)
**CRM4776**	pRS316::*ERV14*-yeGFP	(Sacristan et al., 2013).
**CRM1682**	pRS315::*CHS3*$^{\Delta37}$-GFP	(Rockenbauch et al., 2012)
**CRM2139**	pRS315::$^{\Delta15\text{-}140}$*CHS3*-GFP	(Sacristan et al., 2013)
**CRM2138**	pRS315::$^{\Delta26\text{-}125}$*CHS3*-GFP	(Sacristan et al., 2013)
**CRM2136**	pRS315::$^{\Delta63\text{-}125}$*CHS3*-GFP	(Sacristan et al., 2013)
**CRM1255**	pRS315::$^{\Delta63}$*CHS3*-GFP	(Sacristan et al., 2013)
**CRM3015**	pRS315::*CHS3*$^{1\text{-}490}$-GFP	(Sanchez et al., 2023)
**CRM4393**	pRS315::$^{\Delta126}$*CHS3*$^{1\text{-}490}$-GFP	This work
