# Supplementary material for: Different Proteostasis Mechanisms Facilitate the Assembly of Individual Components on the Chitin Synthase 3 Complex at the Endoplasmic Reticulum
Source: J Fungi (Basel). 2025 Mar 14;11(3):221. doi: 10.3390/jof11030221 (PMC11943272; doi:10.3390/jof11030221)
Supplement: Supplementary file 1 [file jof-11-00221-s001.zip › Supplementary Figures Final.pdf]

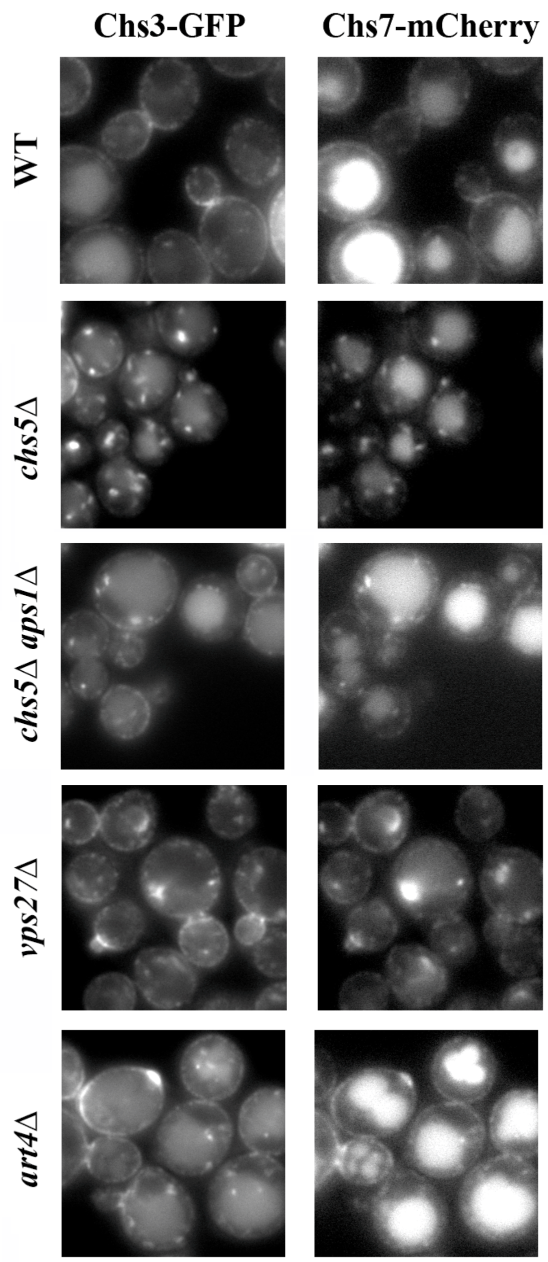

Figure S1. Colocalization of Chs3-GFP and Chs7-mCherry along the secretory pathway. Both proteins were tagged in the chromosome and their localization assessed by fluorescence microscopy; Chs3 was observed in the GFP channel and Chs7 in the mCherry channel. Note that their localization depend on the mutant genetic background. Also, note the almost perfect colocalization of both signals, but how the overall accumulation of m-Cherry in the vacuole is stronger.

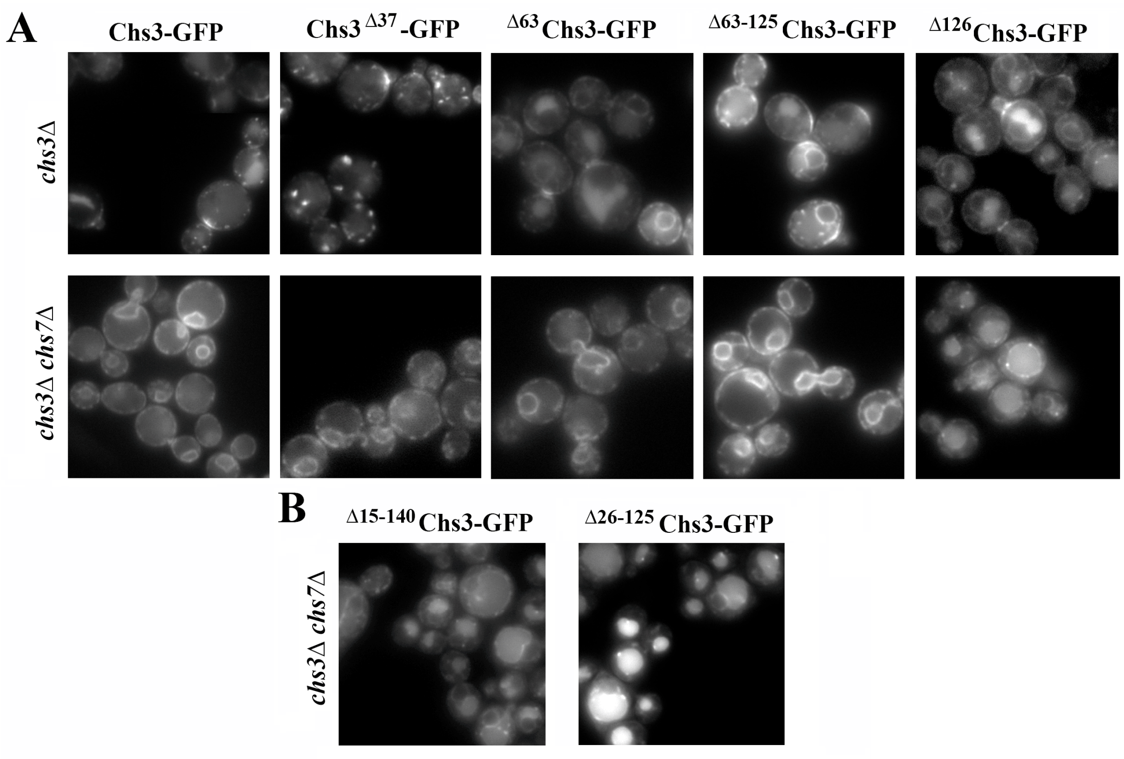

Figure S2. (A) Localization of the indicated versions of Chs3 in the presence or absence of Chs7 as indicated. Note the ER retention of all constructs in the *chs7*Δ mutant except in the case of $^{\Delta 126}$Chs3. (B) Localization of the indicated versions of Chs3 in the absence of Chs7. Note the strong vacuolar signal in both strains.

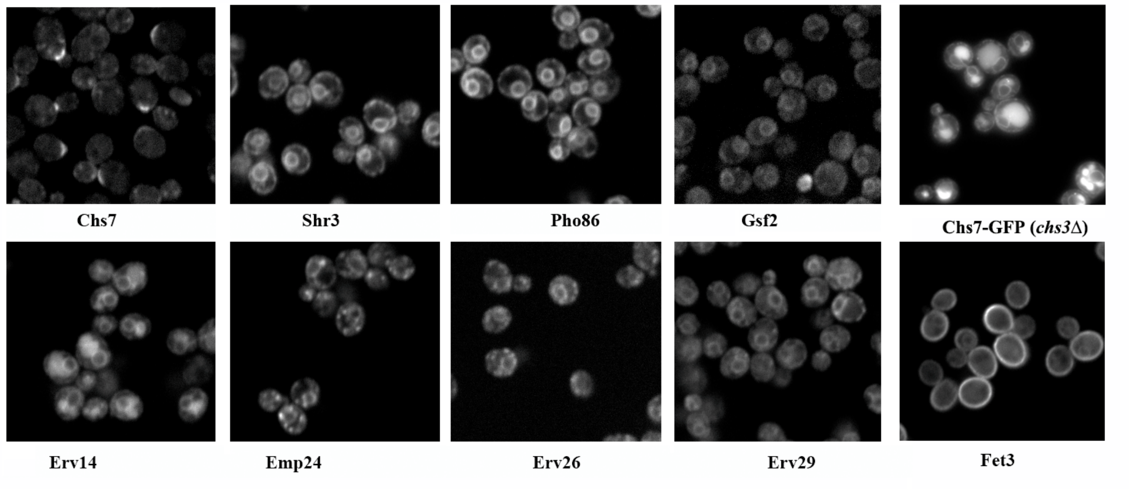

Figure S3. Localization of the indicated proteins. All images were obtained from SGD except for Chs7-GFP (*chs3*Δ), which is from our lab.

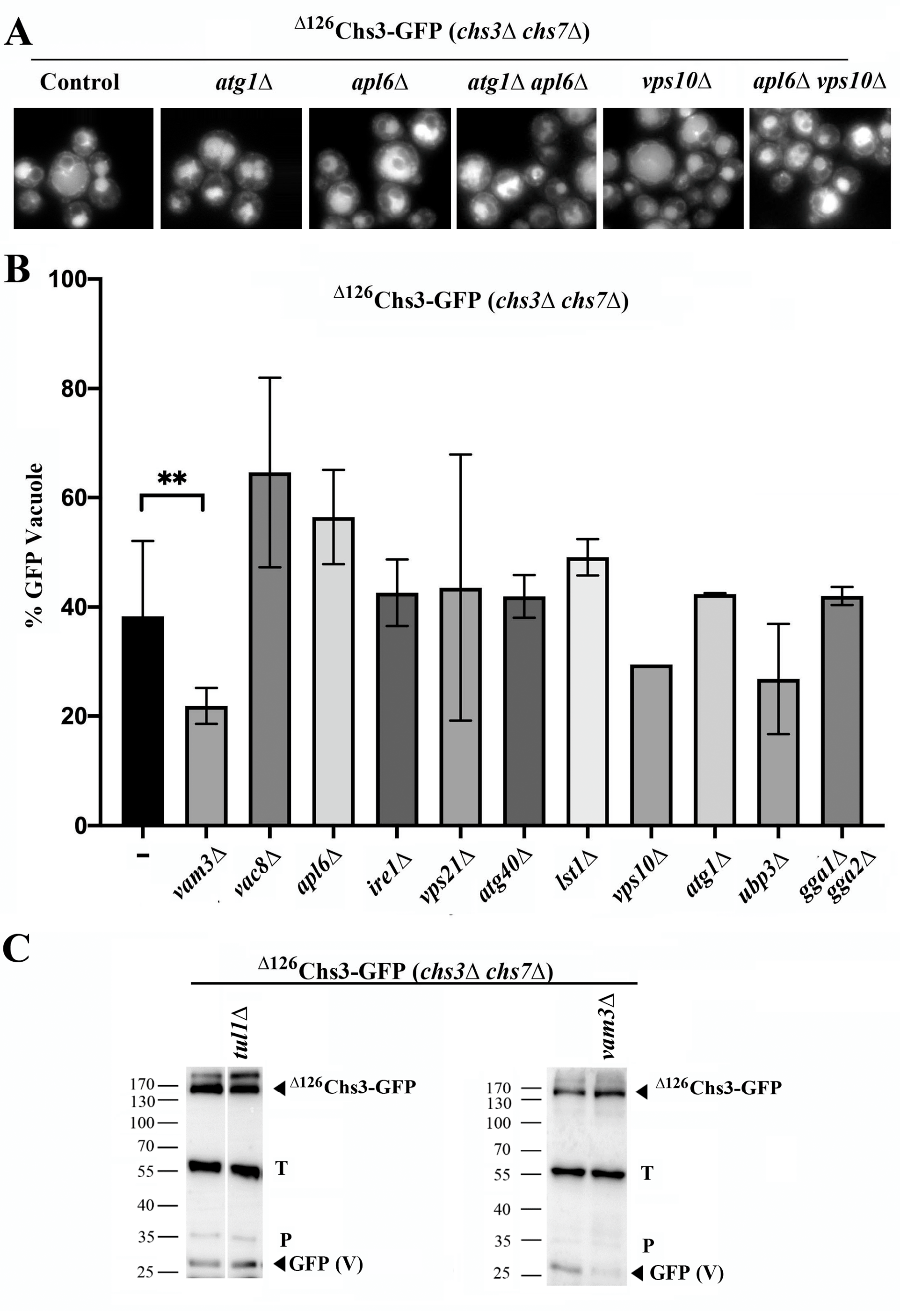

Figure S4. The individual traffic of $^{\Delta 126}$Chs3 to the vacuole. (A) Localization of $^{\Delta 126}$Chs3 in the indicated mutants in the absence of Chs7. (B) Relative amounts of the free GFP band associated with the traffic of $^{\Delta 126}$Chs3 to the vacuole in the absence of Chs7 in the indicated mutants. Note that a significant reduction in the traffic to the vacuole was only detected in the *vam3*Δ mutant. (C) Western blot of $^{\Delta 126}$Chs3-GFP in the indicated mutants showing the amounts of free GFP bands liberated in the vacuole. The absence of Tul1 does not reduce its traffic to the vacuole, but the absence of Vam3 significantly does.

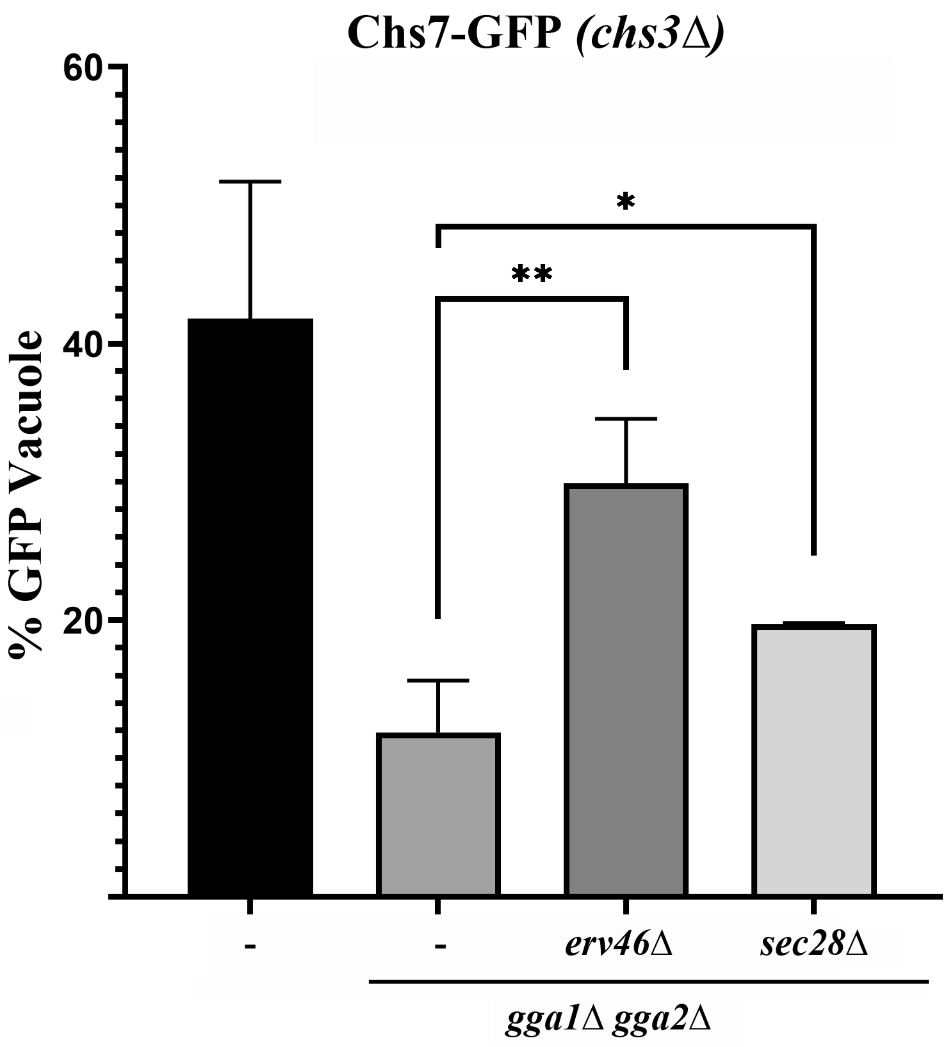

Figure S5. Relative levels of free GFP bands from Chs7-GFP in the absence of Chs3 in the indicated mutants. Note the partial rescue of vacuolar degradation in the *gga1*Δ *gga2*Δ mutant caused by the deletion of different COPI proteins.

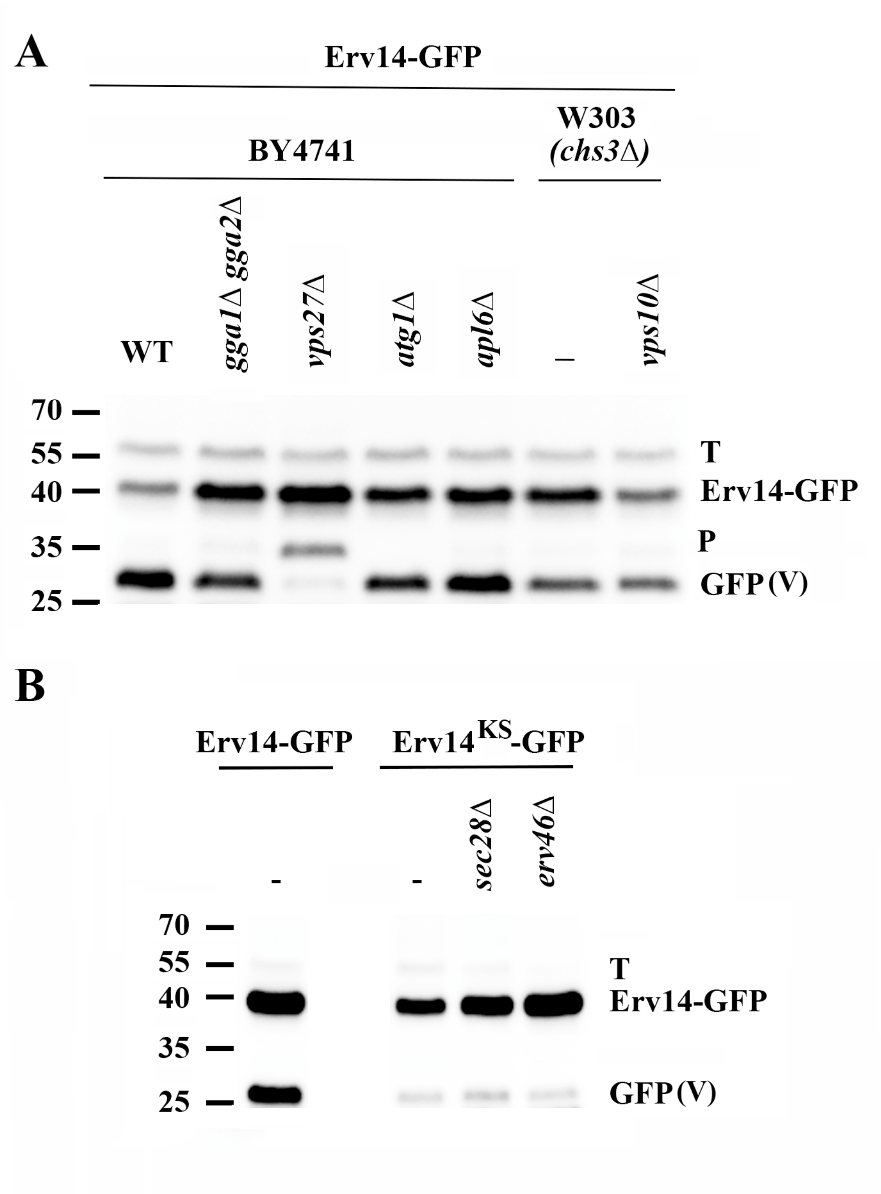

Figure S6. (A) Western blot of the Erv14-GFP protein in the indicated mutants. Note the strong signal of the free GFP band that is strongly reduced in the *vps27*Δ mutant and moderately in the *gga1*Δ *gga2*Δ mutant. Blockage of the traffic to the vacuole in the ESCRT-0 mutant *vps27*Δ facilitates the proteasomal degradation of the protein as deduced from the more intense proteasomal band (P) detected in this mutant. (B) Western blot of the Erv14-GFP protein and its non-functional version, Erv14^{KS}-GFP (Powers and Barlowe, 2002) in the indicated mutants and the wild type strain (-). Note how Erv14^{KS}-GFP is virtually absent from the vacuole in the wild type and in the COPI or COPI-adaptor mutants. GFP (V) refers to the free GFP band and its intensity serves as a marker of vacuolar protein degradation.

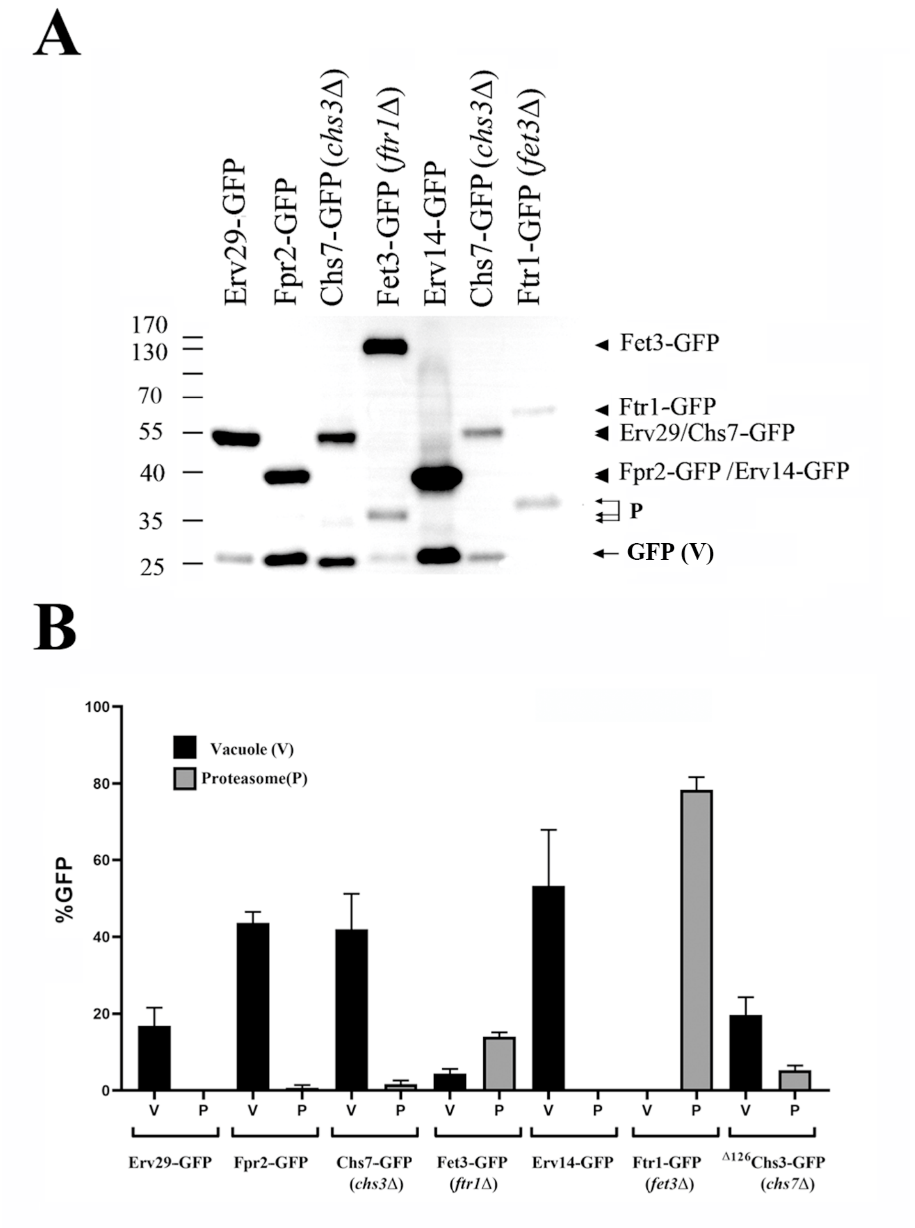

Figure S7. Comparative analysis of the traffic of the individual components of different protein complexes and ER resident proteins. (A) Western blot of different proteins assayed in this work as indicated. Note the different relative amounts of the free GFP (V) bands compared with full-size proteins (arrowheads) and the occasional appearance of a processing band linked to proteasomal degradation (P) for some proteins. (B) Quantitative analysis of the intensity relative levels of GFP bands associated with proteasomal (P) and vacuolar degradation (V). Data of the $^{\Delta126}$Chs3-GFP have been calculated from previous experiments.
